# In Vivo Microplastic Detection With Photoacoustic Imaging

**DOI:** 10.1002/advs.202512152

**Published:** 2026-05-28

**Authors:** Joseph C. Bear, Olumide Ogunlade, Jayvian Mavi, Emily J. Deniszczyc, Daolong Chen, Heeva Javaheri, Paul Beard, Mark F. Lythgoe, Daniel J. Stuckey, P. Stephen Patrick

**Affiliations:** ^1^ School of Life Sciences Pharmacy & Chemistry Kingston University Kingston upon Thames UK; ^2^ Department of Cardiovascular Sciences School of Medical Sciences College of Medicine and Health & Department of Electronic Electrical and Systems Engineering School of Engineering, College of Engineering and Physical Sciences University of Birmingham Edgbaston Birmingham UK; ^3^ Department of Medical Physics and Biomedical Engineering University College London London UK; ^4^ Centre for Advanced Biomedical Imaging, Division of Medicine University College London London UK; ^5^ Hawkes Institute University College London London UK

**Keywords:** imaging, in vivo, microplastics, optoacoustics, photoacoustics, polymers

## Abstract

Microplastics are posing an escalating threat to both ecological systems and human health. Yet, current methods for investigating their bioaccumulation are highly invasive, requiring destructive analysis of ex vivo tissues via mass spectrometry, dye labelling, or Raman microspectroscopy. This limits the study of biodistribution dynamics in preclinical models and human populations, leaving an urgent need for non‐invasive alternatives. Meeting this challenge, for the first time in living tissue, the native optical absorption properties of microplastics are exploited to generate photoacoustic signal – imageable ultrasound emission following thermalisation of pulsed laser light. Distinct optical absorption profiles enable microplastic differentiation from endogenous biological signal sources, long‐term tracking over 2 months in a mouse model, and microscale resolution of particle features verified histologically. This novel approach overcomes previous limitations of optical and nuclear imaging methods relying on fluorescent dyes or radio‐isotopes, going beyond small transparent organisms such as zebrafish or nematodes, and half‐life‐dependent timescales, respectively. By enabling serial monitoring of microplastic biodistribution dynamics, this technique will help interrogate interacting factors such as ingestion route, microplastic shape, size and polymer type – and their effects on accumulation, degradation, clearance, and disease in animal models, and, ultimately, human subjects.

## Graphical abstract

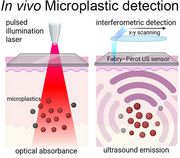

## 1 Introduction

Microplastics are a globally ubiquitous pollutant, found across terrestrial and aquatic ecosystems [1], atmospheric and hydrologic circulatory systems [2, 3], human food chains [4, 5, 6, 7], fluids [8, 9], and tissues [10, 11, 12, 13, 14, 15]. Defined as <5 mm synthetic polymer fragments [16], microplastic release has grown since the invention of man‐made plastics in the late 19^th^ Century. With less than 10% of produced plastic estimated to be recycled [17], environmental microplastic output is projected to reach 80 million tonnes per year by 2040 [18]. Indeed, due to their quantity, prevalence, and persistence in the environment, they are now posited as a future marker or “technofossil” delineating the Anthropocene – the point at which human activities have reached significance on a geological scale [19, 20].

Though the precise risks of microplastic accumulation in the body are unknown, evidence indicates their detrimental impact on fertility and lifespan of various organisms – highlighting potential threats to ecological stability and the safety and sustainability of related food chains [1, 21, 22, 23]. Specific links to human disease are also emerging, with associations found between accumulation in: the liver and its cirrhosis [13], cerebrovascular walls and dementia [15], faeces and irritable bowel syndrome [24], and arterial plaques and risk of stroke and/or myocardial infarction [25]. In response to such concerns, there has been a surge in the development of less environmentally persistent alternatives, including biopolymers and/or biodegradable polymers [26, 27]. However current evidence suggests many of these pose similar, if not greater biological toxicity than established polymer types [28, 29, 30] – necessitating an informed approach to their development and adoption. Yet with multiple interacting factors such as dose, route of ingestion, polymer type and morphology contributing to the difficulty of predicting risk [16], as well as the lack of sufficient research on biodistribution and pathology in relevant cohorts, much still remains unknown on their threat and mechanisms of action [18].

Research in this area is further complicated by the invasiveness of current approaches for identifying microplastics in biological tissue. These are typically biopsy‐based, limiting sampling scope both spatially and temporally, and restricting sample collection to diseased patient cohorts or post‐mortem tissue [31, 32]. This also reduces the practicality of longitudinal preclinical studies and investigation of healthy control participants, leading to calls for better and non‐invasive methods that enable label‐free 3D imaging [33].

Aside from biopsy‐based approaches, microscopic imaging can enable live, non‐invasive imaging in small transparent organisms such as zebrafish or nematodes using particles pre‐labelled with fluorescent dyes [34]. However, these methods do not translate to larger and more optically opaque organisms, including rodents and humans. To this end, radiolabelling has enabled non‐invasive nuclear imaging of the whole‐body distribution of microplastics and exhaust particulates in rodent models [35, 36, 37, 38]. Using PET (Positron Emission Tomography) or SPECT (Single Photon Emission Computed Tomography), these studies provide high‐sensitivity, though low‐resolution data across the body – revealing the effects of size and ingestion route on microplastic biodistribution. However, current nuclear imaging methods require particle pre‐labelling via surface modification, potentially altering their biodistribution in vivo versus unlabelled environmentally representative counterparts. Tracking time is also limited by the half‐life of available isotopes, with previous studies reaching a maximum of 7 days post‐dosing [38]. Label‐free alternatives for microplastic imaging should therefore be prioritized, specifically the development of high‐resolution, high‐sensitivity approaches compatible with tracking of environmentally‐relevant doses over biologically‐relevant time periods.

**FIGURE 1:**
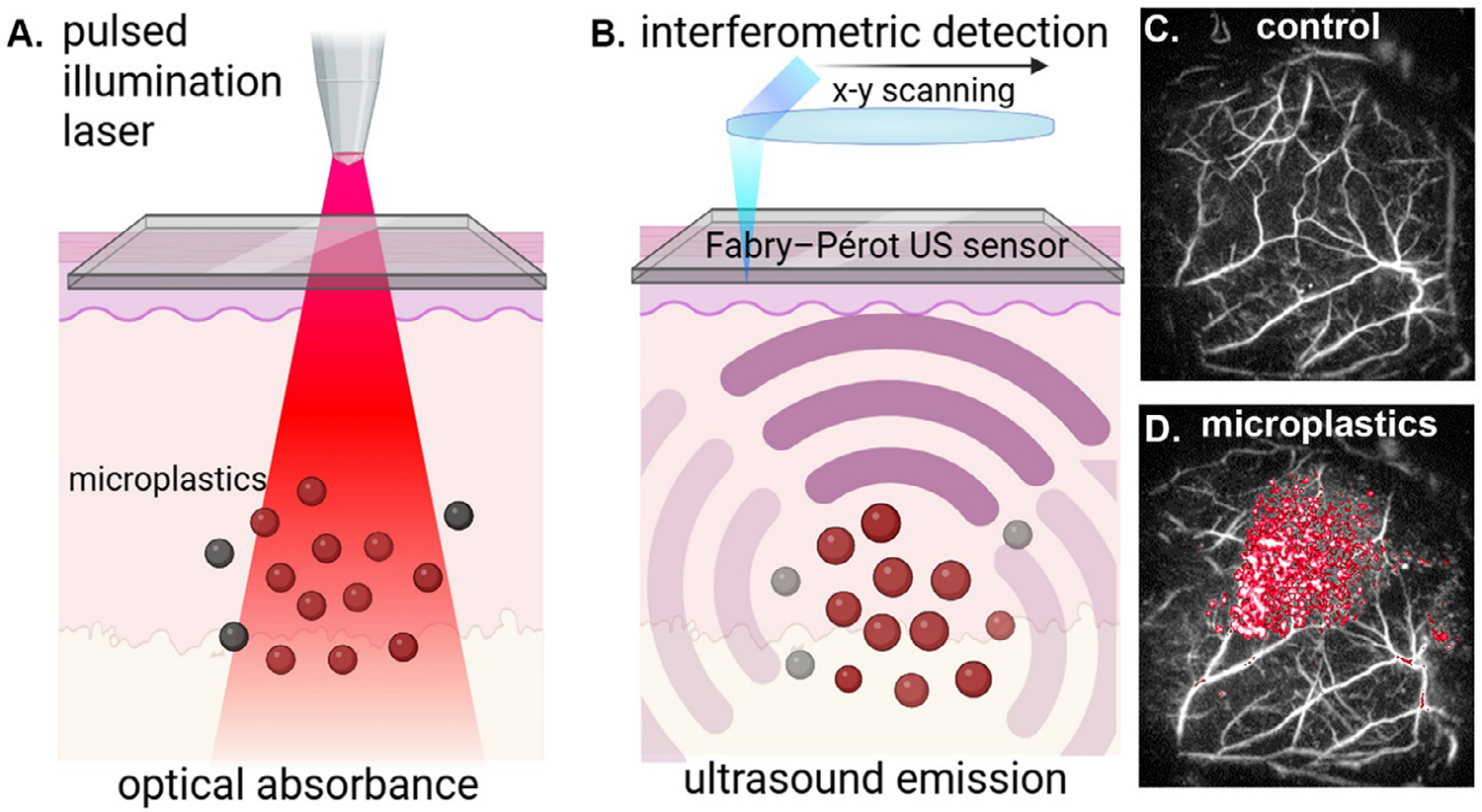
Photoacoustic Imaging of Microplastics. (A) Pulsed laser light illumination of target tissue, followed by (B) laser absorption by microplastic particles, and ultrasound (US) emission and detection. Reconstructed photoacoustic images from (C) control mouse (pre‐injection), and (D) the same region following subcutaneous injection of ∼0.5 mg microplastics (∼125 µm, black polypropylene) showing a blood vessel specific signal collected at 600 nm (grayscale) overlaid with microplastic signal at 680 nm (red).

To address this need, we investigated the native pigmentation of microplastics derived from consumer grade plastics for their ability to produce photoacoustic signal – a phenomenon previously exploited using spectroscopy [39]. imaging and deep‐learning [40, 41] analysis of particles in waste‐water, soil, and household products. Photoacoustic imaging relies on absorption of nanosecond pulsed laser energy by pigments, their subsequent thermalisation, and emission of imageable ultrasound waves (Figure 1A,B). Indeed, as Barthes noted on plastic in 1957, “what best reveals it for what it is, is the sound it gives” [42]. We show in this first proof‐of‐concept in vivo demonstration that photoacoustic imaging provides a novel, high resolution, solution for non‐invasive detection of microplastic particles. This technique circumvents the need to pre‐label microplastics with an exogenous dye or tracer, opening up the possibility of imaging environmentally accumulated material in larger living organisms. Here we show deep imaging in mouse thigh muscle, and evidence of single‐particle resolution. Confirming in vivo findings, we used histology to compare photoacoustic signal with microplastic presence on ex vivo tissue, showing similar size distribution.

Photoacoustic imaging combines the non‐invasiveness of ultrasound imaging with the spectroscopic specificity of optical imaging [43]. Through multi‐wavelength acquisition, we were able to preferentially collect signal from either microplastics or the vasculature, providing an anatomical reference for their location. Our use of an all‐optical Fabry‐Pérot sensor‐based system enabled high sensitivity imaging with high resolution in the tens of microns, resulting in scan times of around 1 min [44]. With the demonstration of comparable performance from clinical photoacoustic devices [45], we hope this provides a route for future research on microplastic accumulation in human studies. Future use of this technology promises to address some of the current gaps in knowledge between microplastic accumulation and related pathology.

## 2 Results

### 2.1 Microplastic Synthesis and Characterisation

**FIGURE 2:**
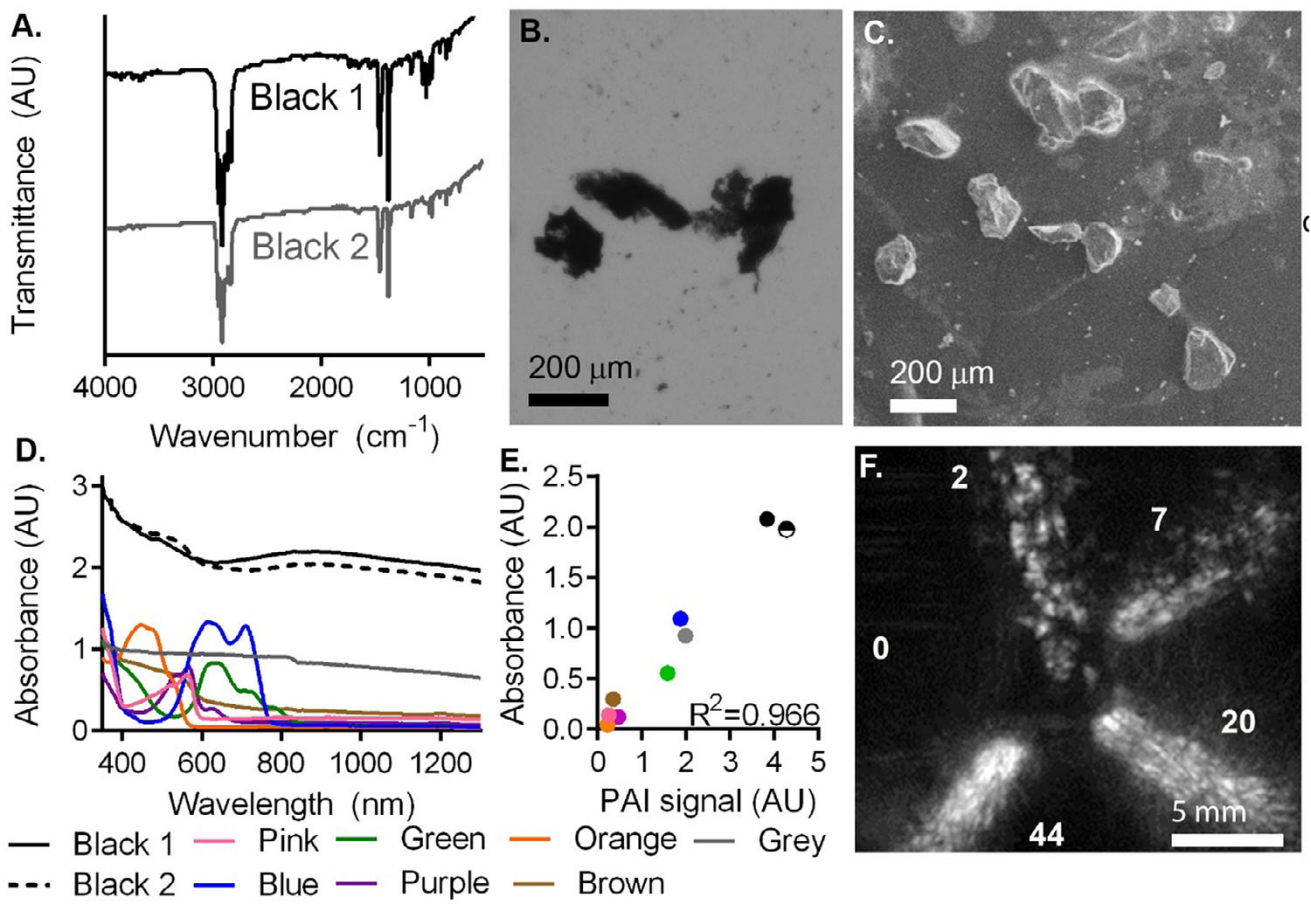
(A) ATR‐FTIR spectra identifying polypropylene as the main constituent of two black microplastic sources used throughout this study. Representative (B) Bright‐field microscopy and (C) Scanning electron micrograph of the 125 µm lower cut‐off size fraction from black microplastic 1. (D) Solid‐state UV–Vis Absorbance spectra of 9 microplastic samples of varying colour and polymer type at 125 µm size fraction, and (E) their absorbance at 680 nm plotted against the mean photoacoustic signal at 10 mg/mL in 2% agar, showing a good correlation. (F) Maximum intensity projection photoacoustic image (680 nm illumination) showing increasing concentrations of microplastic (Black 1, 125 µm sieve size) acquired in microcentrifuge tubes, values showing mg/mL in aqueous suspension.

The majority of published microplastic studies in animal models use commercially available polystyrene microspheres manufactured for research purposes [46]. However, there are concerns over the relevance of this approach, with polystyrene neither among the most abundant microplastics in the environment, or in ex vivo studies of human tissue, where polypropylene, polyethylene, and PET form the majority of identified particles [10, 14, 24]. We therefore developed a new technique to synthesize microplastics from representative consumer plastic sources, using a new sandpaper‐based method to mimic environmental degradation. Size‐controlled fractions were produced by passing each degraded sample through a graded series of vibratory sieves (1 mm, 500, 250, 125, and 45 µm aperture), giving lower and upper size limits to each fraction. Polymer type of the isolated fractions was confirmed using Attenuated Total Reflectance Fourier Transform Infra‐red (ATR‐FTIR), with matching against known spectra of in the Hummel polymer library, confirming the produced samples as polypropylene and polyethylene (Figure 2A; Figures S1–S4). Microplastic size and morphology was characterized using optical microscopy (Figure 2B; Figure S5) and scanning electron microscopy (SEM) (Figure 2C; Figures S6–S10). This gave sizes within the expected range, for example, the fraction passing through the 250 µm sieve and retained in the 125 µm sieve had mean diameters of 166.36 µm ± 68.23 SD for black plastic 1 (BiC Cristal Original pen lids), and 194.39 µm ± 123.12 SD for black plastic 2 (derived from a Winchester laboratory solvent bottle lid; Figures S6–S8), both n = 200. A repeat synthesis of black microplastic 2 (PP) and green microplastic (PE) was similarly characterized via SEM and particle length measurements, with isolated size fractions again measuring within the expected ranges (Figures S9 and S10 and Table S1). To monitor for sandpaper‐related contamination, we quantified silicon content across size fractions for green and black microplastic 1 via energy dispersive X‐ray spectroscopy, giving a negligible average atomic % of 0.15 (Table S2 and Figure S11).

### 2.2 Optical and Photoacoustic Characterisation

Light absorption of target molecules at red to near infra‐red wavelengths is preferable for photoacoustic imaging, due to its lower absorption and scatter by native tissue compared to lower visible wavelengths. We therefore quantified optical absorption for a range of microplastic sources produced as above (Figure 2D), showing the highest absorption of black and grey microplastics across this range (600–1300 nm), encompassing the visible and near infrared (NIR‐I) ranges, and into the NIR‐II window. This was followed by blue and green samples (derived from BiC Kids felt tip pen lids, and polyethylene Robinsons squash bottle lids respectively), which showed absorption in the narrower 600–750 nm range. Based on the higher absorbance and flat optical absorption, the black plastics were selected as the best candidate for imaging, and their PA amplitude spectra was characterized, showing consistency with their measured absorbance spectra (Figure S12). As expected, good correlation of mean photoacoustic signal with optical absorption was found across the whole range of synthesized microplastic samples at both 680 nm (R^2^ = 0.966) and 980 nm (R^2^ = 0.988) (Figure 2E; Figures S12 and S13).

We then investigated the detectability of black microplastics across a range of physiologically relevant concentrations (2 to 45 mg/mL). The minimum tested concentration (2 mg/mL) showed a clear signal compared to a control sample (0 mg/mL) at 680 nm, with increasing signal at higher concentrations (Figure 2F). This spans estimated concentrations in human tissue, with 4.8 mg/g reported in brain samples derived from deceased donors in 2024 [15], while a much higher average of 21.7 mg/g was reported in vascular plaques [25]. Confirming robustness of detectability, high photoacoustic signal stability was also found for both black microplastic samples under prolonged laser exposure (Figure S14). To investigate possible effects of environmental exposure, microplastic absorbance spectra were measured following accelerated ageing with high intensity UV exposure, and incubation for a week in hydrogen peroxide or potassium hydroxide (Figure S15), again showing no change to optical properties. Optical absorbance spectra were also obtained across a range of particle sizes for green (polyethylene) and black 2 microplastics (polypropylene), showing no size dependent changes (Figure S16). This was consistent with photoacoustic data acquired for grey microplastics at 980 nm, which gave no significant correlation between integrated signal intensity, or thresholded pixel count, and size fraction for a controlled weight of material, supporting good detectability independent of particle size (Figure S17).

**FIGURE 3:**
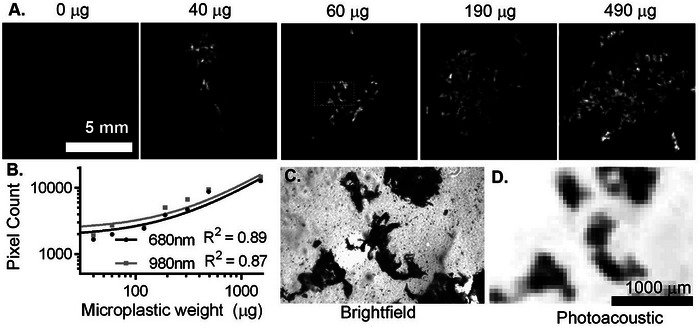
Photoacoustic imaging signal correlates with microplastic quantity and microscale structure in the microgram range. (A) Representative photoacoustic maximum intensity projections (MIP; 680 nm illumination) of black microplastic 2 (250 µm lower cut‐off size fraction) between 0 and 490 µg per field of view. (B) Segmented PAI pixel count versus microplastic weight shows good correlation at 680 and 980 nm (linear regression) between 0 and 1520 µg. Enlarged views of the highlighted region in A acquired with serial (C) Brightfield microscopy and (D) PAI (inverted grayscale to facilitate comparison), with pixelwise correlation analysis showing good agreement (Pearson's correlation coefficient = 0.66).

To assess quantification accuracy on the microgram scale, black microplastics were then weighed out onto adhesive tape and imaged with PAI at 680 and 980 nm (Figure 3). Photoacoustic signal as measured by segmented pixel number correlated well with measured microplastic quantity across this range at both wavelengths (R^2^ = 0.89 and 0.87, respectively (Figure 3A,B), as well as with overall integrated signal (R^2^ = 0.75 (680 nm) and 0.81 (980 nm)). PAI images were then compared to brightfield micrographs of the same sample, with pixelwise correlation analysis confirming the accuracy of PAI in reporting microscale particle structure (Figure 3C,D, Pearson's coefficient = 0.66). Similarly strong brightfield‐photoacoustic correlation was obtained for further samples, confirming the accuracy of this technique (Figure S18).

**FIGURE 4:**
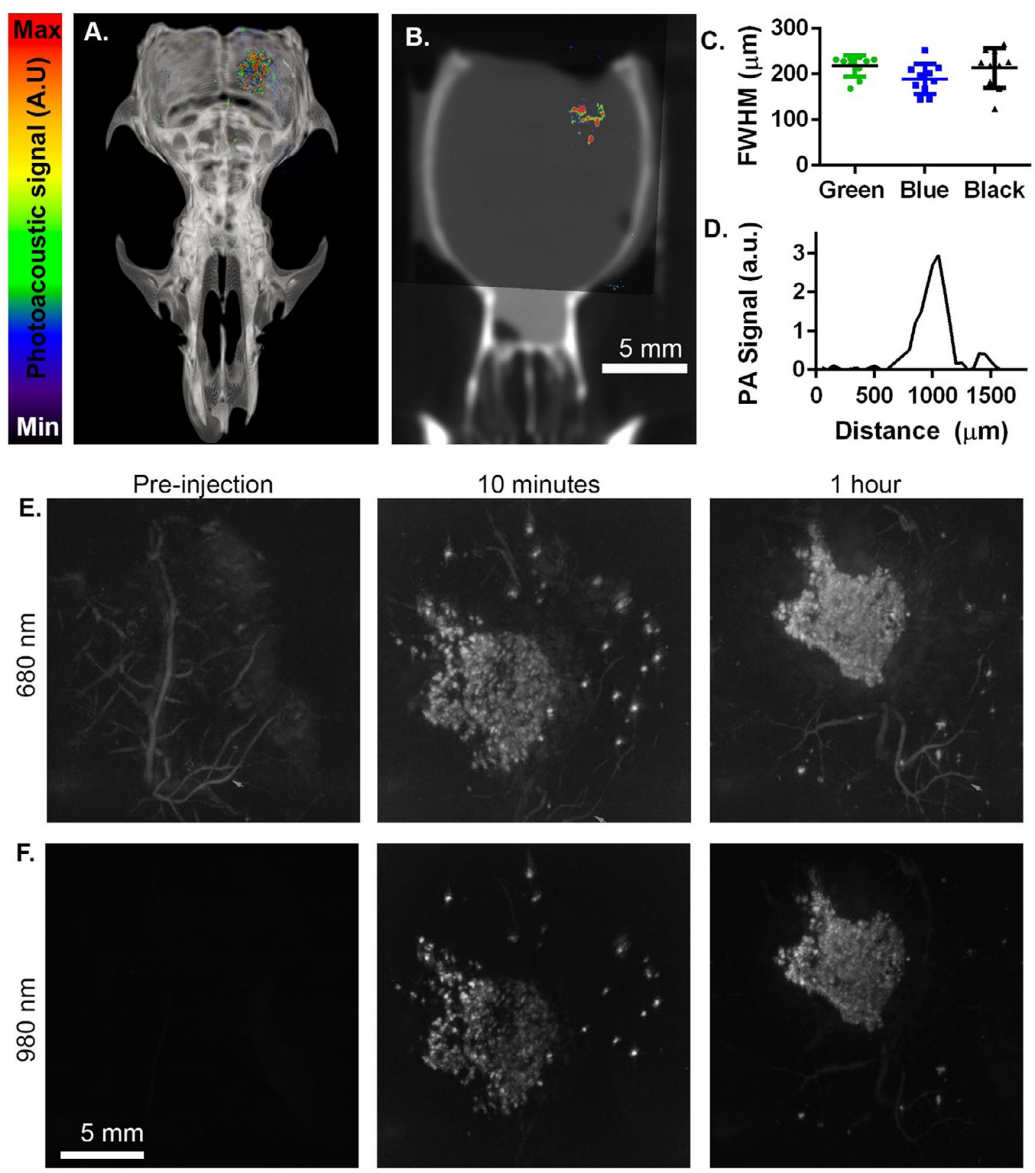
Representative Co‐registered X‐ray CT and Photoacoustic Image showing detection of green microplastics through the rat skull (polyethylene, >125 µm <250 µm, 5 mg/mL) as (A) Maximum intensity Projection and (B) Axial overlay. Microplastics were embedded in a 1% agar bead (right) with a control bead (left) in the contralateral hemisphere being undetectable above background. (C) Particle sizes were measured as full width half maxima (FWHM), showing no significant difference in size between green (PE), blue (PP) and black (PP) samples (No significance, 1‐Way ANOVA, Tukey's Multiple Comparisons, n = 10, *p* > 0.15). (D) Representative FWHM plot for individual PA hyperintensity for Green PE sample.Maximum intensity projections before (pre‐injection), and 10 min to an hour post subcutaneous injection of 0.5 mg of grey polypropylene microplastics (>125 µm <250 µm). Photoacoustic images acquired over 4 averages at (E) 680 nm and (F) 980 nm. The arrow indicates the same vascular feature on each image, provided for anatomical reference.

Before in vivo testing, we next assessed microplastic detectability through the skull. Alginate spheres were dropcast with and without green, blue and black microplastics at a concentration of 5 mg/mL – a concentration previously reported in the human brain [15], and imaged serially with photoacoustic imaging at 680 nm and X‐ray CT in an ex vivo rat skull phantom. PA signal hyperintensities were clearly visible in the microplastic‐containing hemisphere, with no signal above background in the control hemisphere (Figure 4A,B; Figures S19 and S20) for the three microplastic colours investigated. Particle size as measured by full‐width half‐maxima (FWHM) showed no significant difference between colours (Figure 4C,D), further supporting the reliability of the technique. FWHM measurements (n = 10) were also consistent with SEM measurements as above (n = 200, Table S1), showing no significant difference (Green PP, Mann–Witney U test, 2‐tails, *p* = 0.35).

To evaluate short‐term microplastic tracking in vivo, photoacoustic imaging was performed before and after subcutaneous injection of 0.5 mg green (PE), grey (PP), and blue (PP) microplastics in mice. Grey microplastics were clearly visible above the native vasculature 680 nm, with greater specificity for the microplastics versus the vasculature seen at 980 nm (Figure 4E,F). Changes in aggregation were seen between 10 min and 1 h post‐injection, with greater clustering seen at the later timepoint consistent with the particle's hydrophobicity. Similar results were seen for blue (Figure S21) and green microplastics (Figure S22), demonstrating the detectability of a range of microplastic colours in vivo. Supporting the accuracy of in vivo photoacoustic particle size estimates, photoacoustic FWHM measurements (n = 26) and SEM‐based measurements (n = 200) as reported above were compared for the latter, showing no significant difference (Mann–Witney U test, 2‐tails, *p* = 0.24).

### 2.3 Long Term In Vivo Microplastic Imaging After Subcutaneous Injection

**FIGURE 5:**
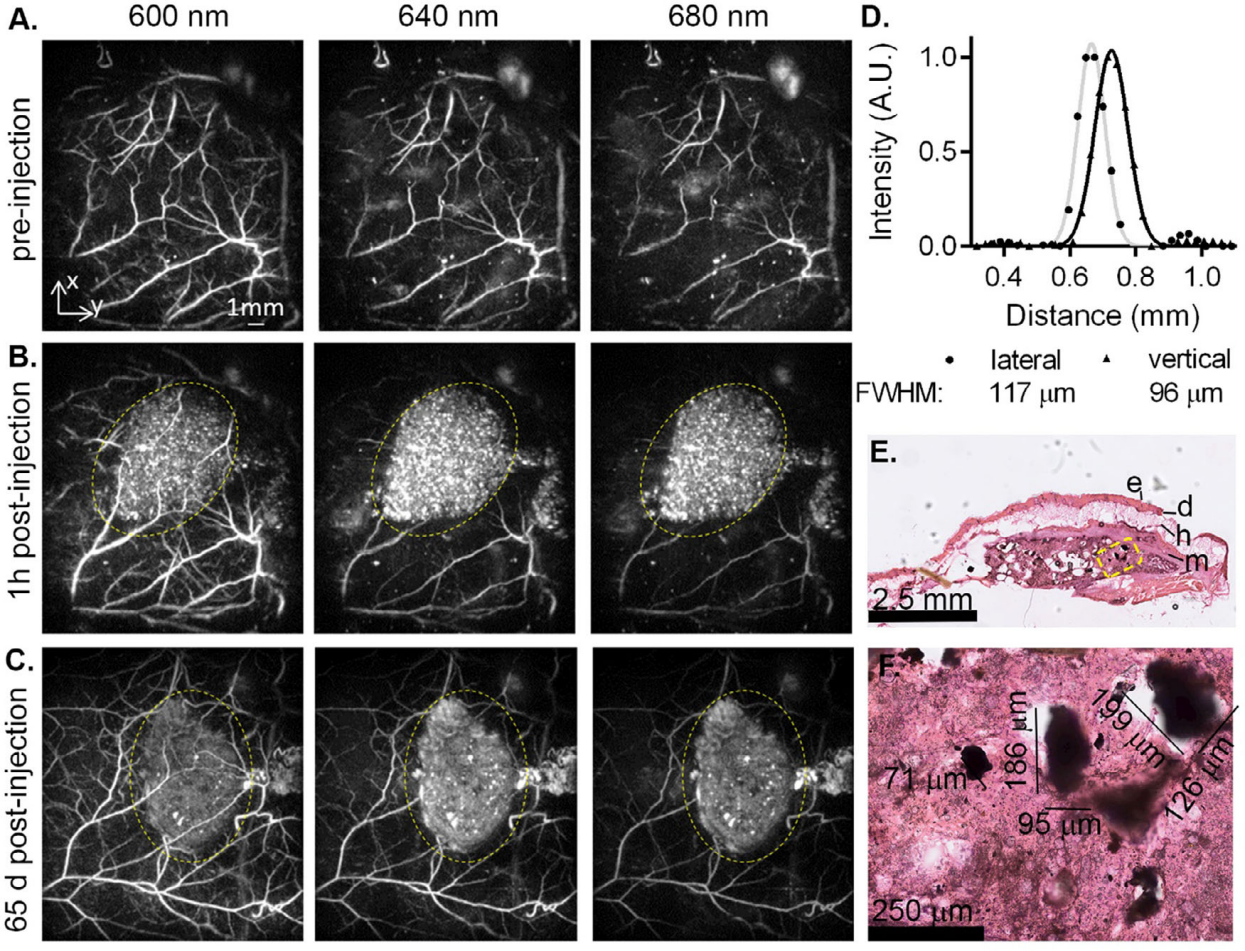
Photoacoustic imaging of subcutaneously‐injected microplastics. Maximum intensity projections (MIPs) taken at 600, 640, and 680 nm of the same site (A) pre‐injection, (B) 1 h post‐injection, and (C) 2 months (65 days) post‐injection of ∼0.5 mg of polypropylene microplastics. (D) Full‐width‐half‐maximum (FWHM) signal profile of a small image feature in the injected region presumed to be a single particle (B, 680 nm), showing dimensions consistent with the size of injected particles. (E) H and E staining of the removed site of injection (e = epidermis, d = dermis, h = hypodermis, and m = muscle), with the highlighted region enlarged in (F), showing size measurements of 3 identified microplastics.

To test the ability of photoacoustic imaging to detect microplastics over extended periods in vivo, imaging was performed before and after subcutaneous injection of 0.5 mg of black polypropylene particles (black microplastic 1, 125 µm sieve, see Figure 2; Figures S2, S7 and S10), using wavelengths between 600 and 680 nm to capture both the and the broadband microplastic absorption, and the order of magnitude change in haemoglobin‐related absorption over this range [47]. Pre‐injection images again clearly showed the native vasculature (Figure 5A), which appears brightest at 600 nm, consistent with the strong absorption of blood at this wavelength. A large and intense region of additional signal was visible at the site of injection at both 1 h (Figure 5B), and 2 months post‐injection (Figure 5C), showing better contrast versus vasculature at 680 nm consistent with the flat absorption peak of the black microplastics and the greater distance from the known haemoglobin peaks. Also consistent with the assumption that the detected signal was derived from the injected microplastics, the lateral and vertical full‐width half maximum (FWHM) dimensions corresponded well with those of the injected individual particles as measured with SEM and microscopy (Figure 5D). To further validate this, the tissue surrounding the injected region was removed after two months and cryo‐sectioned for histology. The injected mass of particles was found in the sub‐muscular region on hematoxylin and eosin (H+E) stained sections (Figure 5E), in line with their appearance on the photoacoustic images. Microplastics identified on histological sections were analysed for size, giving a mean length of the longest dimension of 148.6 µm ± 56.8 SD (n = 25), comparable to values obtained from the material pre‐injection (Figure 4F; Figure S7 and Table S1; *p* = 0.34 2‐tailed Mann–Witney U‐Test).

### 2.4 Microplastic Detection in Deeper Muscle Tissue

To investigate microplastic detectability in deeper tissue, a similar bolus was injected into the hind flank muscle, with imaging performed pre and post injection to control for endogenous signal. In this case, a sample of black microplastic 2 was used (polypropylene, 125 µm sieve size, see Figure 2; Figures S2, S8–S10), to show compatibility with different consumer plastic sources.

**FIGURE 6:**
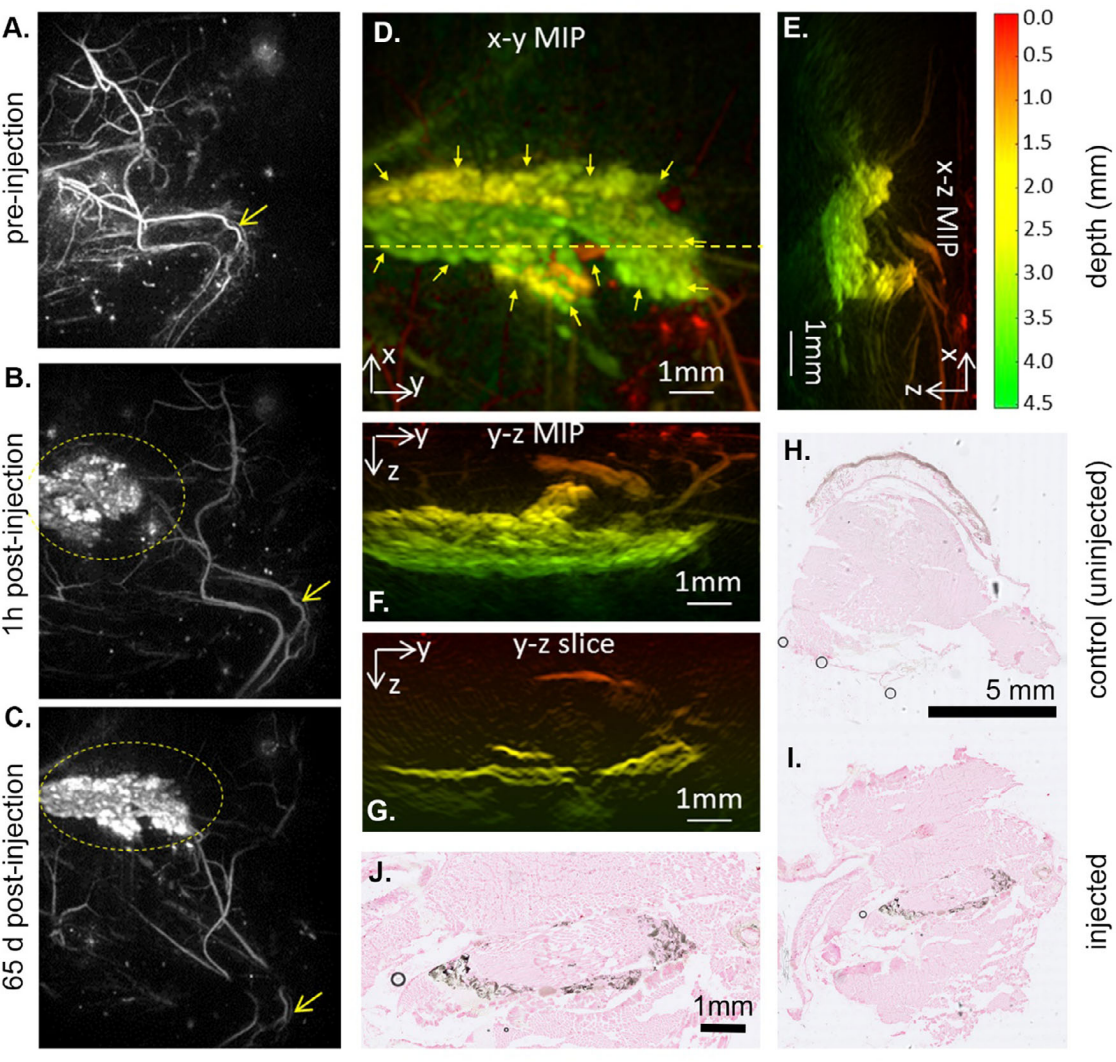
Photoacoustic imaging of microplastics in the hind flank muscle. Maximum intensity projections of the same region at 680 nm at (A) pre‐injection, (B) 1 h post‐injection (0.5 mg microplastics), and (C) 2 months (65 days) post‐injection. Yellow arrow points to the same reference vein to show image orientation. Colorized MIPS showing signal depth of the microplastic bolus in (D) the *x*‐*y* plane, (E) the *x*‐*z* plane (F) the *y*‐*z* plane, and (G) of a single slice in the *y*‐*z* plane (50 µm thickness). Nuclear fast red‐stained section of (H) uninjected control flank muscle, and (I) microplastic‐injected flank muscle, with (J) Enlarged region showing microplastic injection site matching the photoacoustic slice shown in G.

Pre‐injection images again clearly showed the native vasculature (Figure 6A), and the additional region of hyper‐intensity at 1 h and 2 months post‐injection (Figure 6B,C; Figure S23). On the other hand, the contralateral flank showed no change in signal intensity before or after injection of a carrier‐only solution, showing only the native vasculature (Figure S24). Reconstructions from the 2‐month scan were then colour‐coded to visualize signal depth below the skin, and maximum intensity projections were produced in the *x*‐*y*, *x*‐*z*, and *y*‐*z* planes (Figure 6D–F). This showed a clear signal from microplastics up to 4 mm below the surface, within the site of injection in the leg muscle. To confirm the presence of microplastics in the regions indicated on the photoacoustic image, leg muscles were removed from the injected and uninjected flanks. As expected, microplastics were not visible in the uninjected muscle on histological sections (Figure 6H), however a clear injection track was visible within the injected muscle (Figure 6I,J). This closely matched the signal obtained from in vivo imaging in depth and shape (Figure 6G,I,J), consistent with the microplastics being the photoacoustic signal source. Again, size analysis of the microplastics was performed on histological sections, giving a length of the longest observable dimension of 163.5 µm ± 53.43 SD (n = 23) comparable again to values obtained from the material pre‐injection (Figure 6J; Figure S8 and Table S1; *p* = 0.38, 2‐tailed Mann–Witney U ‐Test).

## 3 Discussion

This work demonstrates the first non‐invasive detection of microplastics without exogenous labels in a live mammalian system. To do this, we exploited the photoacoustic effect, whereby absorption of pulsed nanosecond laser light by native microplastic pigments resulted in the emission of imageable ultrasound waves. This gave high resolution across a 2 × 2 cm window up to depths of ∼4 mm in vivo, showing microplastic distribution in mouse thigh muscle with a fast scan time of around 1 min per wavelength. This builds on previous work investigating microplastic distribution in mouse models using nuclear imaging techniques [35, 36, 37, 38], though here we circumvent the need to modify the particle surface, and extend the duration of tracking beyond the 1 week previously demonstrated up to 2 months.

With polypropylene being one of the most widely found microplastics polymer types in human tissue [10, 14, 24], our ability to detect it in a mouse model clearly illustrates the technique's potential for future studies linking accumulation dynamics to pathological processes and the onset of disease. Here, the ability to visualize microplastics using a separate modality complements existing non‐invasive imaging techniques, including MRI, nuclear imaging, and bioluminescence for visualising related pathological processes including fibrosis [48, 49] inflammation [50, 51], oxidative stress [52], and changing tissue metabolism [53]. Though photoacoustic imaging seems optimally suited to the detection of black microplastics, blue, green, and grey samples were also shown here to have sufficient absorption for in vivo detection at tissue‐penetrating wavelengths. As it is the pigment and not the polymer that is directly detected, we expect wide compatibility across polymer types. Though few studies report the colour of microplastics found in human tissue, necessitating further research, over half of those examined in the placenta were described as blue or dark [10], suggesting future applicability of this technique beyond rodent models and basic research. Similarly, grey and black microplastics together were found to constitute 78% of microplastics in a recent study on dairy products [54]. While transparent microplastics would not be detectable with this method, and those with pigments absorbing at lower wavelengths are less suited to in vivo imaging due to poorer tissue penetration, future research on the statistical distribution of plastic colours found in human tissue would allow extrapolation of tissue content from the imageable fraction. And while a further limitation of this technique is the inability to resolve nano‐plastics (being below the spatial capabilities of the detector design), we found absorbance spectra, and PAI signal to be comparable between size fractions here. We will therefore investigate in future work whether nano plastics are also detectable at similar weight‐to‐volume ratios as the microplastics successfully detected here.

Sensitivity of detection of microplastics was found here to be high, enabling clear visualisation across the full range 3 orders of magnitude of concentrations tested (40µg‐40 mg/mL). To put this in context with amounts found in human tissue, 4.8 mg/g has recently been reported in brain samples derived from deceased donors in 2024 [15], while a much higher average of 21.7 mg/g was found in vascular plaques [25]. Though recent concerns have been raised that methodological deficits in studies using pyrolysis gas chromatography–mass spectrometry (Py‐GC–MS) for microplastic detection have led to over‐estimations, this further highlights the need for improved methods. Though we used a controlled injection bolus of microplastics for this proof‐of‐principle study, the potential of investigating more realistic accumulation through environmental exposure is supported by the high sensitivity of detection demonstrated here. In terms of microplastic size, our ability to detect particles in vivo with mean diameter below 150 µm supports future use in oral exposure studies, with microplastics in this size range reported to cross the intestinal epithelium [55]. Also supporting future use in tracking microplastics dosed under more environmentally‐representative conditions ‐we were able to detect particle features well below the mean reported diameter of ∼275 µm for microplastics in the environment [16]. Here, we confirmed the accuracy of PAI reporting on microstructure using brightfield microscopy, showing high levels of correlation via pixelwise comparison. For the detection of yet smaller particles, photo‐acoustic microscopy systems have been reported to enable real‐time, hand‐held imaging with resolution down to 7 µm [56]. Further gains in sensitivity might also be achieved using multi‐spectral acquisitions, spectral unmixing [57, 58], and/or deep‐learning [40, 41] to reduce confounding signal from endogenous pigments.

Promising to extend microplastic detection to the brain and internal organs in future studies, whole‐body, high‐resolution preclinical imaging has now been achieved using multiple Fabry–Perot detectors to obtain tomographic images [59] – overcoming the restricted field of view achieved by the single‐sensor system used in this study. While in the clinic, photoacoustic devices have also been developed by a number of research groups and manufacturers [43] – enabling larger‐scale imaging across the human body, including the brain, gastrointestinal system, tongue, hand, foot, joints, and breast, with depths up to 5 cm [43, 45, 60]. With some of the strongest evidence for microplastic risks to human health involving accumulation in vascular plaques [25], the development of clinical intravascular photoacoustic technology offers a means to go beyond the depth penetration limits of external devices such as those used here [61]. Beyond clinical investigation, workplace/occupational monitoring of microplastic exposure and uptake is also an active area of research. Multiple links have been found between exposure in plastics/ synthetic textiles factories and biomarkers of liver function [62], inflammation, lung function [63], and rates of gastrointestinal cancer [64]. Here, the ability to combine non‐invasive tissue‐specific microplastic accumulation measures with current biomarker and outcome monitoring would provide a means to better understand such occupational risks and their associated pathological mechanisms.

In conclusion, we present a fast, non‐invasive, high‐sensitivity, high resolution approach to detect unlabelled microplastics in living tissue. We demonstrate this over environmentally representative concentration ranges, biologically relevant timespans, and in superficial and deeper tissue in a living mouse model. This technique removes prior limitations associated with ex vivo biopsy‐based techniques, and in vivo label‐based imaging approaches, promising to open up new routes of research into the behaviour of microplastics in the body and their role in disease.

## 4 Materials and Methods

### 4.1 Microplastic Synthesis

Microplastics were derived from a range of commercial sources including black biro pen lids (BiC Cristal Original pen lids; black 1), the black lid of a 2.5L Winchester solvent bottle (black 2), and multiple colours (grey, pink, blue, black, orange, purple and brown) from felt tip pen lids (BiC Kids), and from green squash bottle lids (Robinsons, various flavours).

Microplastics were prepared by placing a square sheet of sandpaper at the bottom of a glass petri dish before covering the sheet in ethanol (VWR, 99% denatured with methanol). Bulk microplastics were then sanded, taking care to replace the sandpaper frequently to avoid degradation of the sandpaper surface and silica contamination of the resultant microplastics. The resultant microplastic particles were air‐dried overnight and sieved using a descending order of mesh sizes, specifically 1 mm, 500, 250, 125, and 45 µm, using a vibratory sieve shaker (AS 200, Retsch). Samples were then retained and labelled with the sieve size in which they were retained, setting an approximate lower limit on the size of their smallest dimension.

### 4.2 Microplastic Characterisation

Microplastic particles prepared as above were characterized using a variety of techniques including: scanning electron microscopy (SEM), attenuated total reflectance Fourier transform infrared (ATR–FTIR) spectroscopy and solid‐state UV/Vis spectroscopy (diffuse reflectance).

SEM images were taken on a Zeiss Evo50 (Oxford Instruments, Cambridge, UK) scanning electron microscope, with micrographs obtained at an acceleration voltage of 20 kV.

ATR‐FTIR measurements were acquired on a Nicolet is5 FTIR (Thermo Scientific) with an iD7 ATR accessory over a wavelength range of 400–4000cm^−1^. Polymers were indexed against the Hummel polymer database included in the OMNIC software (Thermo Scientific).

Absorbance spectra were obtained with a UV–Vis spectrometer (Shimadzu UV‐2600i) with diffuse reflectance accessory over a 200–1400 nm wavelength range, using the Kubelka‐Munk transformation on the acquired diffuse reflectance data. Samples were prepared by taking the 125 µm sieved microplastic samples and sandwiching it between 2 glass slides and securing with tape. Spectra were corrected against a blank microscope slide.

For in vitro photoacoustic methods, see Supporting Information.

### 4.3 Photoacoustic Correlation With Bright‐Field Microscopy

Microplastic particles (Black plastic 1, sieve size 500 µm) were attached from a clean surface to adhesive tape (Scotch, Clear Tape, 25 mm) using moderate pressure. Imaging regions were marked using a grid around the sample using a black marker pen to enable cross‐reference between modalities. Tape samples were then placed shiny, non‐adhesive side down on the PAI sensor (deepColor, Lightecho‐R) on top of a 2 mm layer of ultrasound gel (Anagel), with further gel applied on top of the adhesive side of the tape. Images were acquired at 50 µm resolution using 4 averages at 680 nm illumination, and reconstructed using the manufacturer's software with acoustic autofocus. After photoacoustic imaging, the tape was secured shiny side down on a microscopy slide, and coverslipped using the ultrasound gel as a mounting medium, before brightfield acquisition using a light microscope (EVOS, Thermofisher) fitted with a 4x objective. PAI MIPs were rendered using ImageJ software using a linear colour scale, and correlation analysis performed using the Coloc 2 plugin.

### 4.4 Animal Work and Histology

Female BALB/C mice were purchased from Charles River and used at 3 months old at the point of implantation. Mice were housed five per individually ventilated cage, at 21°C with normal day/night cycles. All animal studies were licensed under UK Home Office regulations and approved by the UCL Biological Services Ethical Review Committee, with all regulations in compliance with UCL experimentation guidelines and regulations. For subcutaneous injection, the mouse was imaged before and after injection of a subcutaneous bolus containing a total of 0.5 mg polypropylene microplastics (suspended in a 100 µL solution of 60% GranuGEL (Convatec), 40% PBS as used previously [65]) derived from the BIC Cristal Original pen lids. For the hind flank muscle injections, the mouse was imaged before and after injection of the right flank with 0.5 mg of black polypropylene microplastics (derived from the solvent bottle lid, see above) suspended in a 100 µL solution of 60% GranuGEL (Convatec), 40% PBS). The left flank was injected with a solution of the 60% GranuGEL, 40% PBS lacking the microplastics as a control. Implanted tissue was dissected out at 10 weeks post‐implantation, and freeze‐embedded in optimal cutting temperature media (OCT, Thermo Fisher). Samples were cryosectioned (Leica, Bright 5040) at 10 µm onto glass slides (Thermo‐Fisher, Superfrost Plus), and left to air dry at room temperature. Tissue was then fixed on the slides in 4% buffered formaldehyde solution for 5 min, before washing in phosphate‐buffered saline (×3). Nuclear fast red staining was done using a 5 min incubation in nuclear fast red solution (Sigma Aldrich), before rinsing in distilled water, dehydration via ethanol series, and mounting and cover slipping using Histomount (National Diagnostics). H and E staining was done using an automated H and E protocol using a Tissue‐TEK DRS autostainer (Sakura), with mounting and coverslipping as above. All slides were imaged using a NanoZoomer S360MD slide scanner (Hammamatsu) using a 20 x objective, and viewed using NDP viewer software (Hammamatsu).

### 4.5 In Vivo Photoacoustic Imaging

Mice were anesthetized with 2% isoflurane in 100% O_2_, and maintained under anaesthesia during imaging. In vivo PA images were acquired using a small‐animal scanner based on a tunable optical parametric oscillator (OPO) laser system (Quanta Ray Pro‐270/premiScan; Newport Spectra Physics/GWU Lasertechnik) and a planar Fabry‐Pérot (FP) ultrasound sensor, which has previously been described [44, 66, 67]. Briefly, the OPO generated 7 ns optical excitation pulses for widefield illumination, and consequent generation of PA signals within the illuminated volume via optical absorption [47]. The PA signals are detected by a FP ultrasound sensor, which comprises of two dichroic mirrors separated by a thin (22 µm thick) polymer spacer [44]. PA signals incident on the sensor modulates the optical thickness of the polymer spacer, which produces a corresponding change in the reflectivity of the mirrors. The change in sensor reflectivity was read out by detecting the time‐varying intensity modulation of an interrogation beam, which is focused onto the sensor surface, corresponding to the incident PA signal. In this study, eight interrogation beams were simultaneously scanned across a sensor area of ∼17.8 × 15.8 mm, in steps of 106 µm, to acquire ∼25 000 PA signal waveforms in ∼1 min. 3‐D PA images were reconstructed from the acquired waveforms using a time reversal algorithm [68], which was implemented using an open‐source MATLAB toolbox k‐wave [69], with a correction for acoustic attenuation in tissue based on time‐variant filtering [70]. PA images were acquired at three excitation wavelengths; 600, 640 and 680 nm, to distinguish microplastic from background vasculature, based on their distinct optical absorption spectrum. The maximum excitation pulse energy was 14 mJ, delivered in a 2 cm diameter beam; thus the maximum fluence at the surface of the mouse skin was 4.5 mJ/cm^2^. The reconstructed images were displayed as 2‐D horizontal and vertical maximum intensity projections (MIPs) using a logarithmic intensity scale.

To obtain an indication of the smallest microplastic that could be visualized, horizontal and vertical profiles were obtained across a small microplastic in the PA image. The full‐width‐half maximum (FWHM) of the profiles was determined from a Gaussian function fitted to the data, to approximate the spatial resolution.

### 4.6 Statistical Analysis

All statistical analyses were performed in GraphPad Prism 6.

## Conflicts of Interest

PB is a shareholder of DeepColor Imaging SAS. The remaining authors declare no conflicts of interest.

## Supporting information




**Supporting File**: advs75504‐sup‐0001‐SuppMat.docx.

## Data Availability

The data that support the findings of this study are available from the corresponding author upon reasonable request.
