# Supplementary material for: In Vivo Microplastic Detection With Photoacoustic Imaging
Source: Adv Sci (Weinh). 2026 May 28;13(41):e12152. doi: 10.1002/advs.202512152 (PMC13335746; doi:10.1002/advs.202512152)
Supplement: Supplementary file 1 — Supporting File: advs75504‐sup‐0001‐SuppMat.docx. [file ADVS-13-e12152-s001.docx]

**Supplementary Information** to

***In Vivo* Microplastic Detection with Photoacoustic Imaging**

Joseph C. Bear^1^, Olumide Ogunlade^2,3^, Jayvian Mavi^4^, Emily J. Deniszczyc ^4^, Daolong Chen^4^, Heeva Javaheri^1^, Paul Beard^3^, Mark F. Lythgoe^4^, Daniel J. Stuckey^4^, P. Stephen Patrick^4*^

1. School of Life Sciences, Pharmacy & Chemistry, Kingston University, Kingston upon Thames KT1 2EE, United Kingdom

2. Department of Cardiovascular Sciences, School of Medical Sciences, College of Medicine and Health & Department of Electronic, Electrical and Systems Engineering, School of Engineering, College of Engineering and Physical Sciences, University of Birmingham, Edgbaston Birmingham B15 2TT, United Kingdom

3. Department of Medical Physics and Biomedical Engineering, University College London, London, WC1E 6BT, United Kingdom

4. Centre for Advanced Biomedical Imaging, Division of Medicine, University College London, London, WC1E 6DD, United Kingdom

*peter.patrick@ucl.ac.uk

**Supplementary Methodology**

**Photoacoustic Spectroscopy**

The PA amplitude spectra of the two plastics with highest optical absorbance were measured using a custom designed PA spectroscope (1). The samples contained in a cuvette were irradiated by the output of a tuneable excitation laser at wavelengths between 550 – 680nm in steps of 10nm (covering 600-680nm subsequently used for in-vivo imaging). The generated PA signals were detected using a broadband PVDF detector. The PA spectrum was obtained by plotting the normalised peak-to-peak signal amplitude as a function of wavelength, corrected for variation in excitation pulse energy.

**Signal stability measurements**

The thermal effect of laser fluence on microplastic signal stability were assessed by measuring the PA signal amplitude generated by microplastic in a custom cuvette, as a function of the number of 680nm excitation pulses, for a continuous train of 10,000 pulses at 30Hz. A PA signal was recorded after every 50 pulses, the recorded signal representing an average of the 50 generated signals.

***In vitro* Photoacoustic (PA) characterisation**

An 2% agar phantom was produced using a gel comb, with 10 mg/mL microplastic sample (Black 1, Black 2, Grey, Pink, Purple, Blue, Green, Brown, Orange) all at 125 µm size fraction loaded into each well in a volume of 40µL, then topped with a further 1cm of 2 % agar. This was imaged at 680 nm and 980 nm with a Vevo LAZR-X system and MX201 transducer using a 3D Ultrasound and Photoacoustic acquisition. Region of interest analysis was performed in VevoLab software (Visualsonics) using B-mode ultrasound signal to identify each well, and mean PA value extracted for each identically-sized region of interest.

**Microplastic Ageing**

Ageing experiments took place on a sample of 250 um black microplastics derived from a black poly(propylene) solvent lid (synthetic method described previously). 110 mg of microplastics was weighed out and placed in a vial with either one of the following: 10 mL sulfuric acid (ACS reagent, 95.0-98.0%, Merck Ltd.), 10 mL 4M aqueous potassium hydroxide (from KOH pellets, Fisher Scientific Ltd.) or 10 mL aqueous hydrogen peroxide (35% w/w aq. soln., stab., Fisher Scientific Ltd.). Vials were sealed save for a small hole in the top of the vial lid to prevent gas build up. Samples were stirred at room temperature for 1 week, before dilution with 100 mL deionised water and filtration and drying *in vacuo*. A further sample was spread onto a watch glass and placed under a 254 nm UV lamp (Analytik Jena, 6 watt, 0.16A) in a darkbox at 18cm distance from the bulb, and irradiated for 3 days at room temperature.

**Rat skull phantom imaging**

Green (polyethylene), black, and blue (both polypropylene) microplastics were suspended in 1% w/v sodium alginate solution at 5mg/mL and dropcast into a 300mM calcium chloride crosslinking solution, and incubated 3 minutes under stirring before manual separation and washing. Control beads were aslo prepared using the same method, without microplastics. Prior to imaging each rat skull was filled with gel to mimic the acoustic properties of tissue (Anagel), and 1 control and one microplastic containing bead was placed into each hemisphere. Skulls were sequentially imaged at 680, 850 and 980 nm using a commercial photoacoustic system (deepColor, Lightecho-R), at 12mJ laser power, before imaging at 70 and 90 kV using a microCT system (Quantum GX2, Revvity) at 76 µm resolution and 86mm FOV, and Al 0.5 mm + Cu 0.06 mm filters. Photoacoustic and X-ray CT images were coregistered in 3D Slicer software (Fedorov et al., 2012) using manual rigid alignment. Particle diameter was estimated in Fiji/ImageJ (2) via a custom ImageJ macro script utilising line-intensity profiles drawn across the centre of putative individual microplastic particles. For each straight-line ROI, the intensity profile was extracted and baseline-corrected by subtracting the local background intensity, calculated as the median intensity of the first and last 10% of the line profile. The peak of the baseline-corrected profile was identified, and the full-width at half-maximum (FWHM) was calculated as the distance between the left and right half-maximum crossings. FWHM was reported in spatial units using the image pixel size (50μm⨯50μm).

**Additional *in vivo* imaging**

Mice (BALB/C, male, 4 weeks old, Charles River) were injected subcutaneously in the rear flank with green (polyethylene), grey, or blue (both polypropylene) microplastics (0.5mg, >125µm <250µm) in a 50µL volume of 1.8% w/v sodium alginate. Photoacoustic imaging was performed before and after injection at 12mJ laser power using 980 and/or 680 nm using a commercial system (deepColor, Lightecho-R), and 4 averages. Images were reconstructed using acoustic autofocus at 50 µm resolution and visualised as maximum intensity projections in ImageJ software (NIH.org).


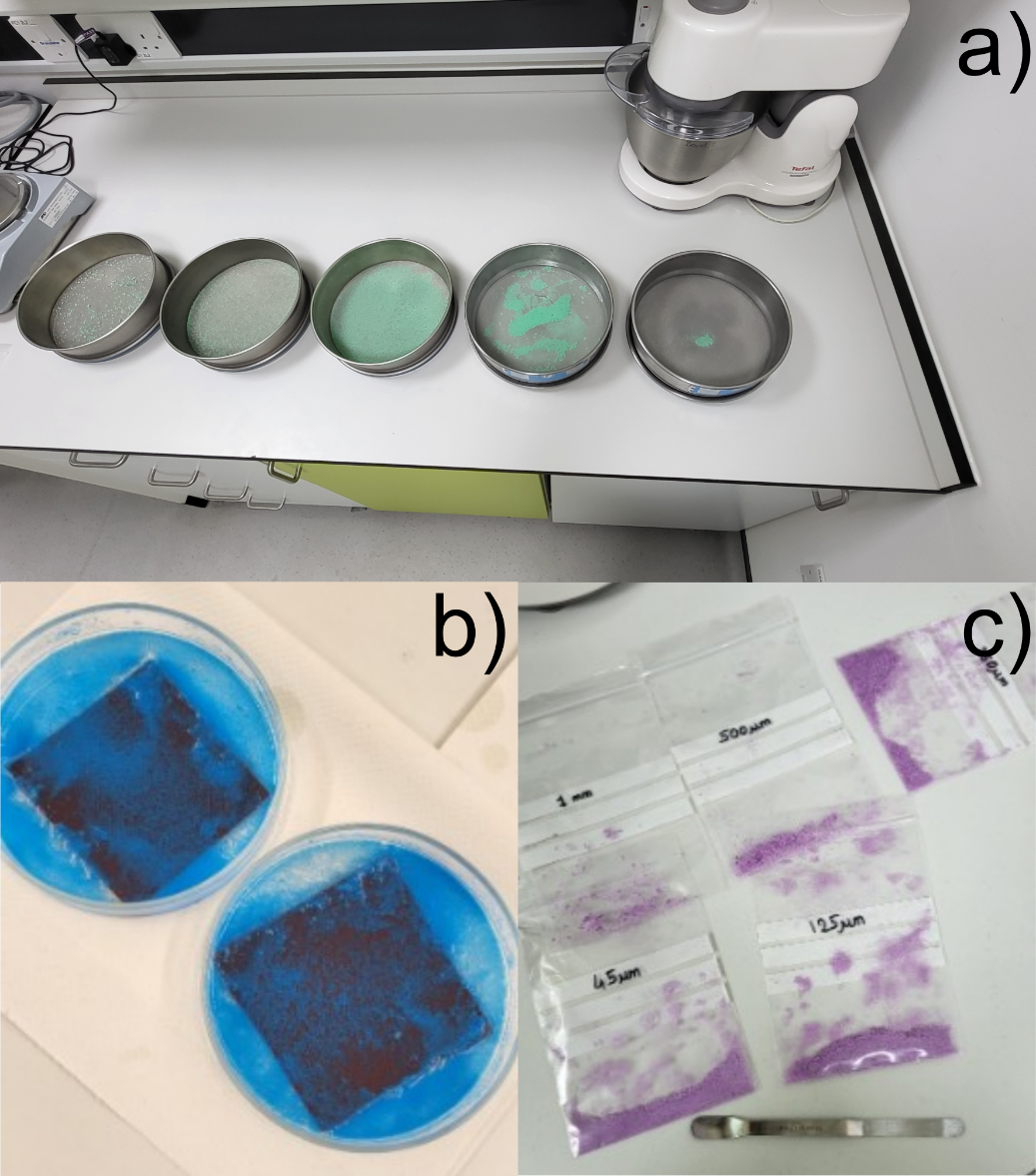


**Figure S1**: a) Photograph of green microplastic particles passed through 1 mm, 500 μm, 250 μm, 125 μm and 45 μm sieves. From left to right, the 500 μm, 250 μm, 125 μm, 45 μm and bottom pan are shown. b) shows the immersed square of sandpaper in ethanol after a blue felt tip pen casing was sanded and c) different size fractions post-sieving of purple microplastic particles in sample bags.


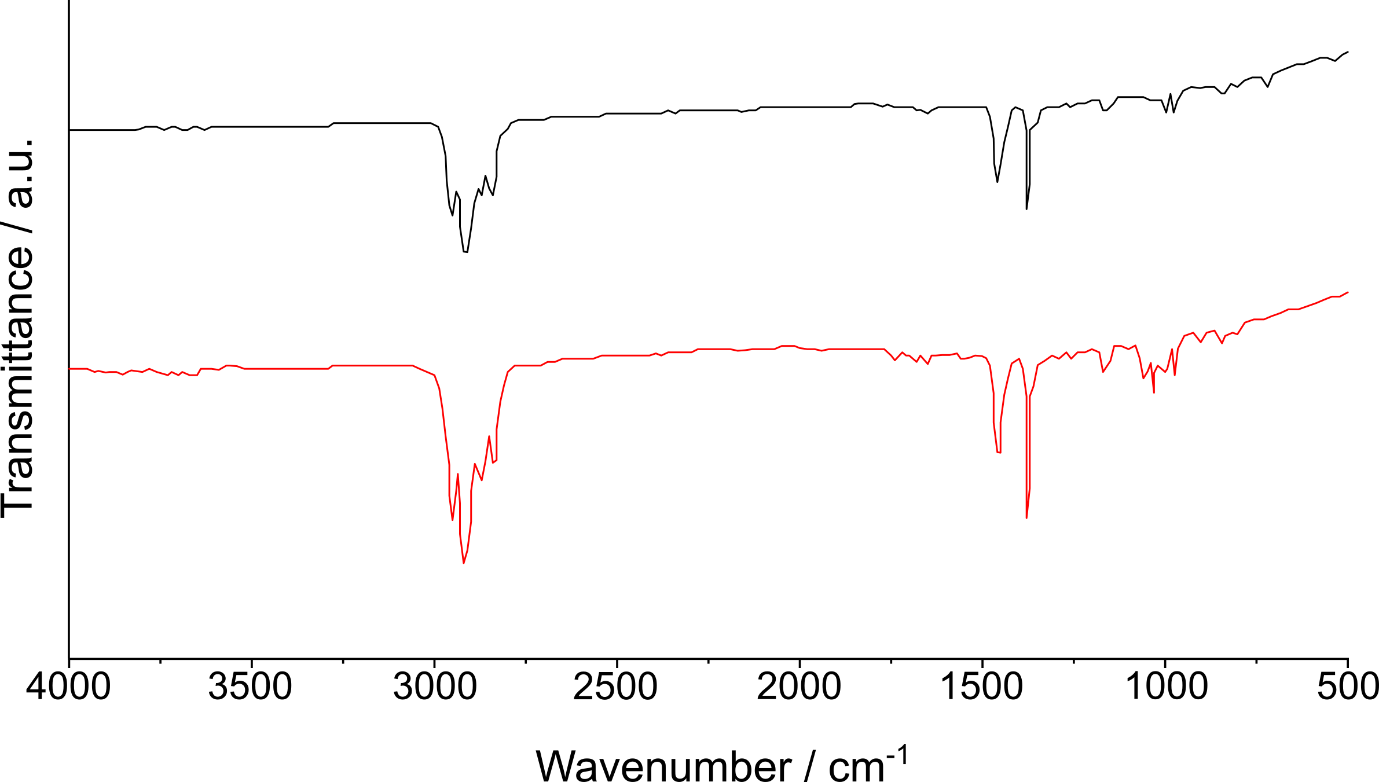


**Figure S2**: Fourier transform infrared (FTIR) spectra of: (black) microplastic particles obtained from solvent Winchester lids (black microplastic 2) and (red) microplastic particles obtained from black biro lids (black microplastic 1). Peak positions correspond to atactic poly(propylene), obtained from the Hummel polymer library available on the OMNIC® software. (ṽ = 1164 cm^-1^ (C–H wagging), 1376 cm^-1^ (CH_3_ symmetric bending), 1457 cm^-1^ (CH_3_ symmetric bending), 2839 cm^-1^ (CH_3_ stretching), 2866 cm-1 (CH_3_ stretching), 2919 cm^-1^ (CH_3_ asymmetric stretching), 2954 cm^-1^ (CH_3_ asymmetric stretching).


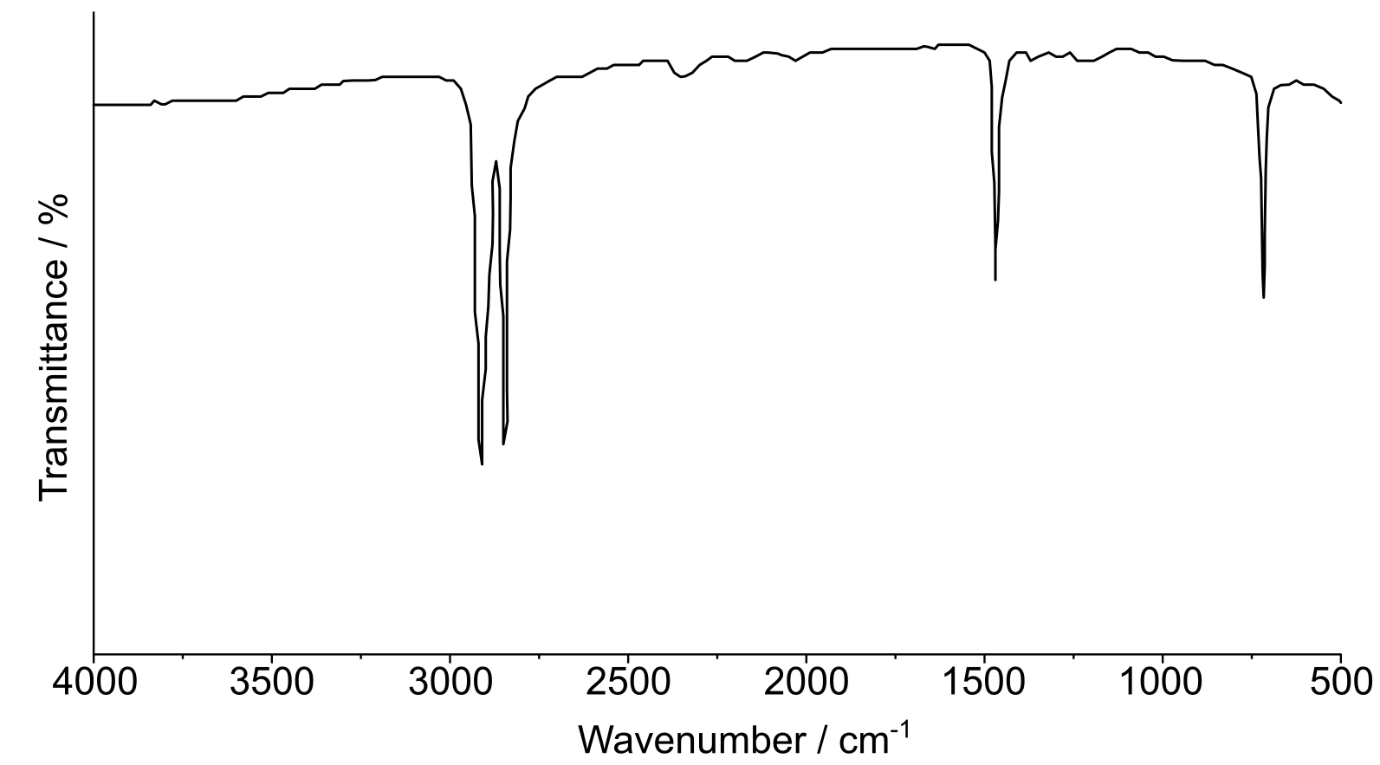


**Figure S3:** Fourier transform infrared (FTIR) spectra of microplastic particles obtained from green Robinsons® squash bottles. Peak positions correspond to poly(ethylene), obtained from the Hummel polymer library available on the OMNIC® software. (*ṽ* = 725 cm^-1^ (C–H rocking), 1468 cm^-1^ (CH_2_ symmetric bending), 2851 cm^-1^ (CH_2_, symmetric C–H stretch), 2921 cm^-1^ (CH_2_, asymmetric C–H stretch).

**
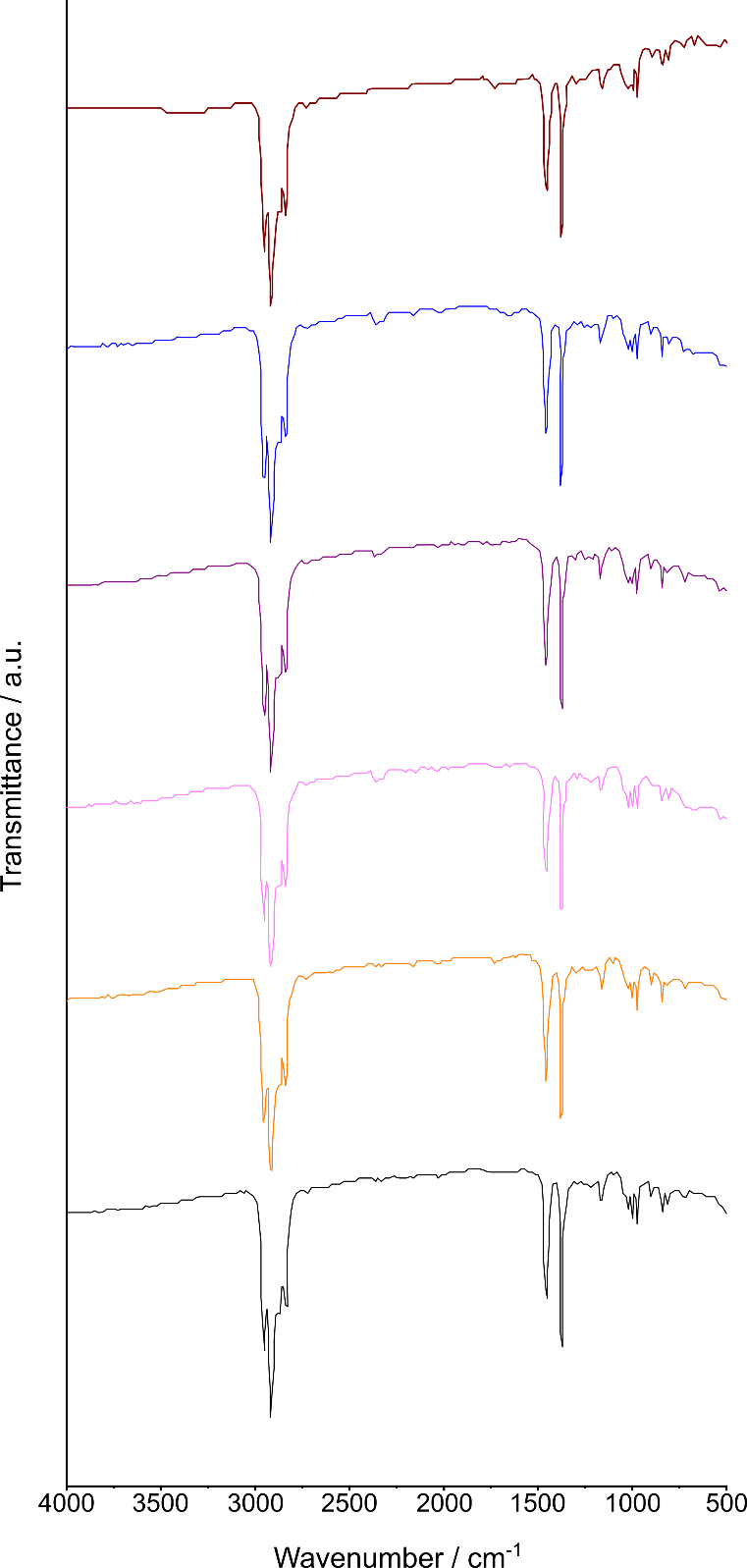
**

**Figure S4:** Fourier transform infrared (FTIR) spectra of microplastic particles obtained from the lids of different colour felt-tip pens. Colours of the spectral lines correspond to the visual plastic colours. Peak positions all correspond to poly(propylene), obtained from the Hummel polymer library available on the OMNIC® software. (*ṽ* = 973 cm^-1^ (C–H rocking), 997 cm^-1^ (C–H rocking), 1164 cm^-1^ (C–H rocking), 1376 cm^-1^ (CH_3_ symmetric bending), 1457 cm^-1^ (CH_3_ symmetric bending), 2839 cm^-1^ (CH_3_, C–H stretch), 2919 cm^-1^ (CH_2_, asymmetric C–H stretch) and 2954 cm^-1^ (CH_3_, asymmetric C–H stretch).


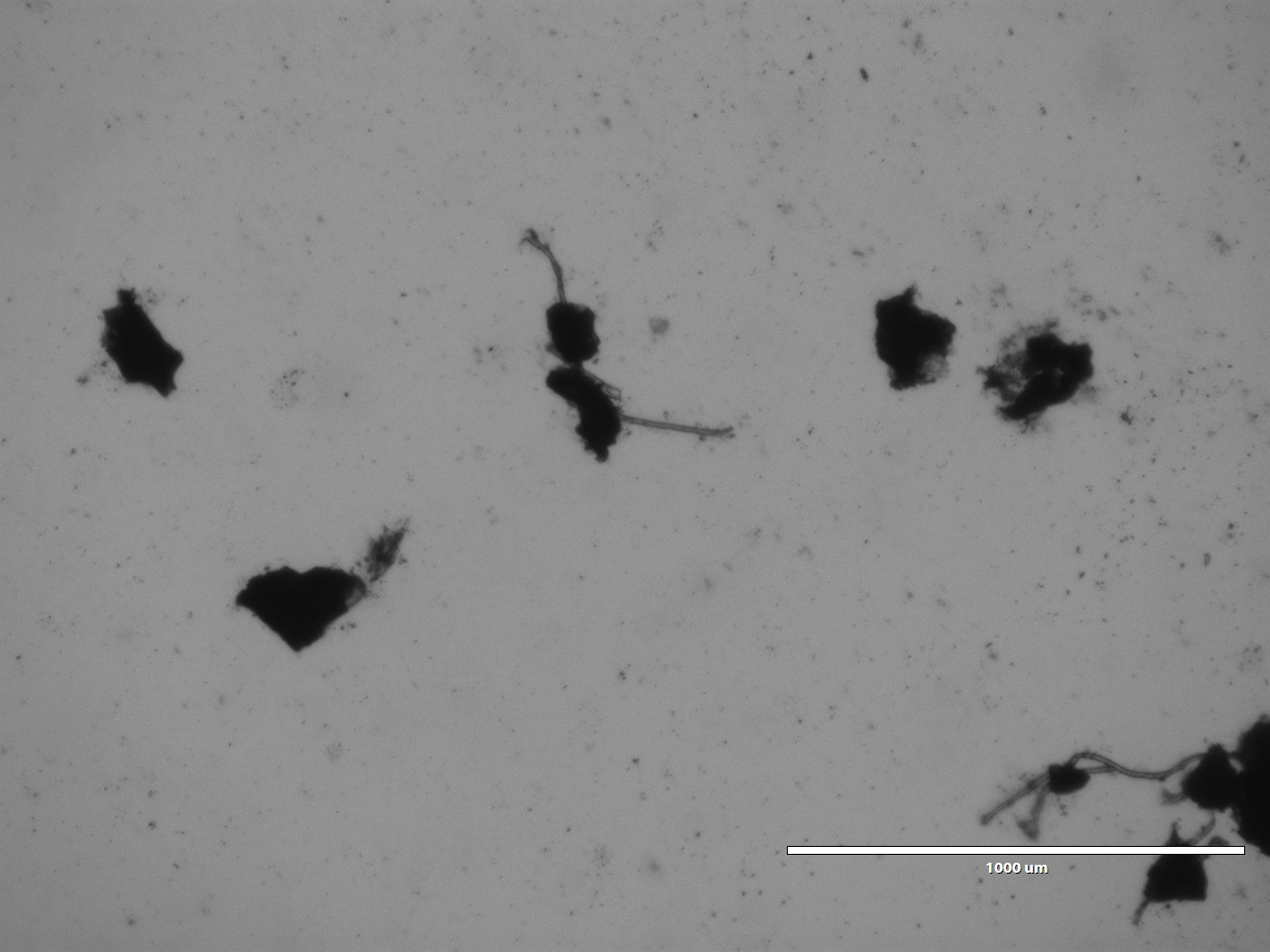
**Figure S5.** Representative bright-field microscopy image showing Black microplastic 2 (125µm size fraction) taken on EVOS™ XL (Thermo Fisher) at 40x magnification.

**
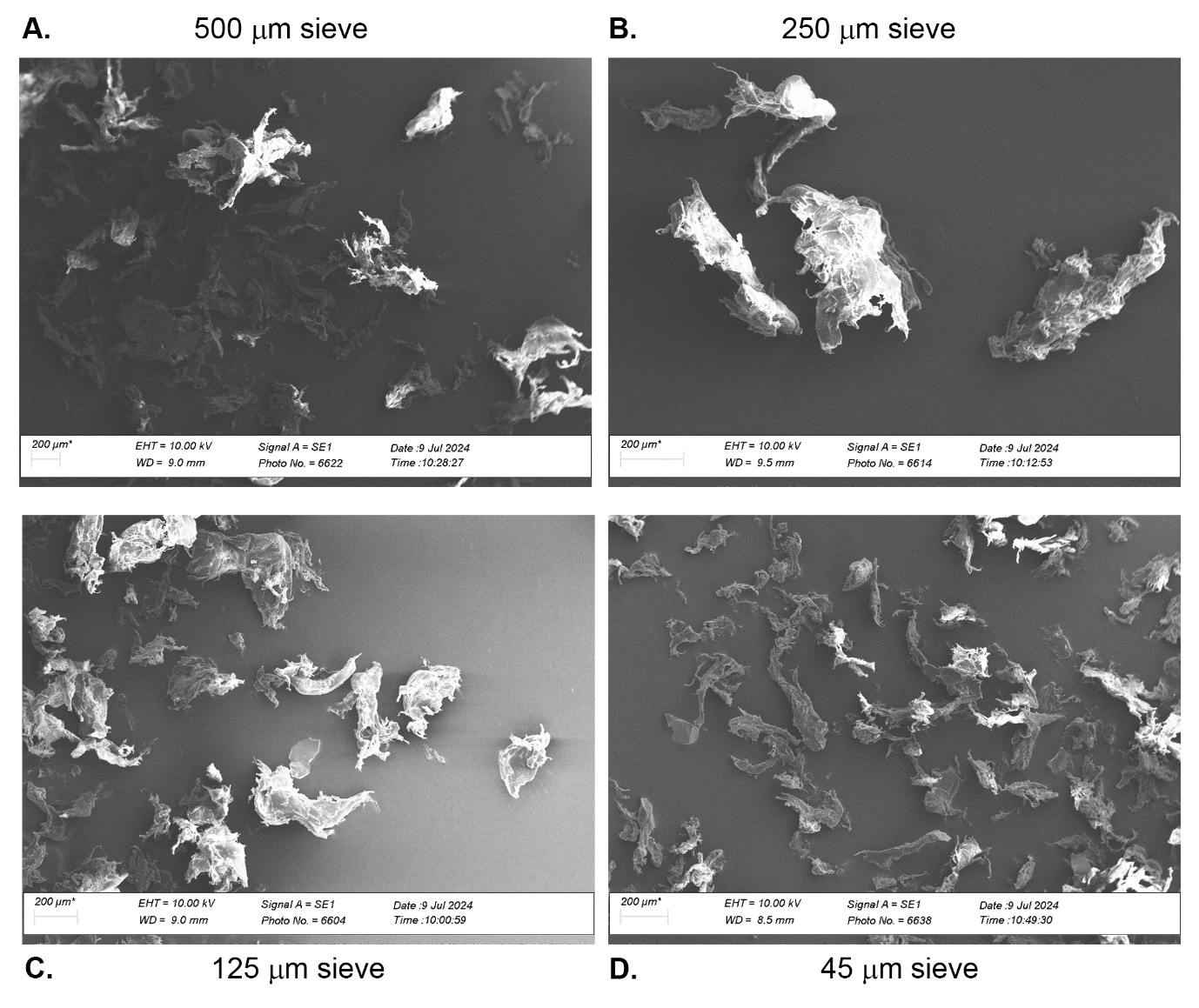
Figure S6.** Representative scanning electron micrographs showing microplastics derived from BiC felt tip pen lids (grey) retained in **A.** 500 µm **B.** 250 µm **C.** 125 µm and **D.** 45 sieves.


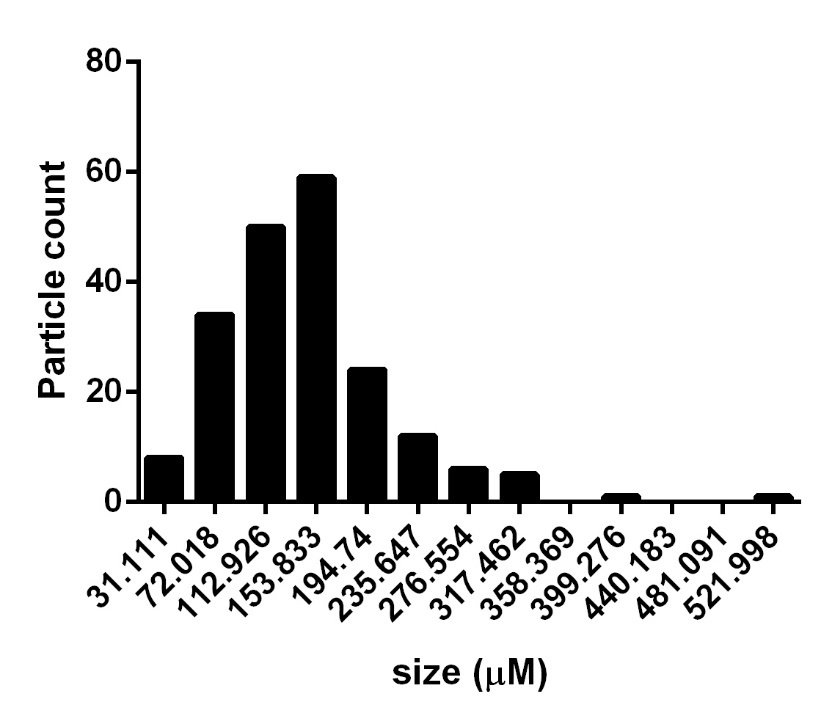


**Figure S7.** Histogram showing size distributions of the 125µm lower cut off fraction of black microplastic 1 (derived from BiC biro pen lids). Mean size 166.36 µm ±68.23 SD. Sizes were measured manually on SEM images of >200 individual particles using ImageJ software.


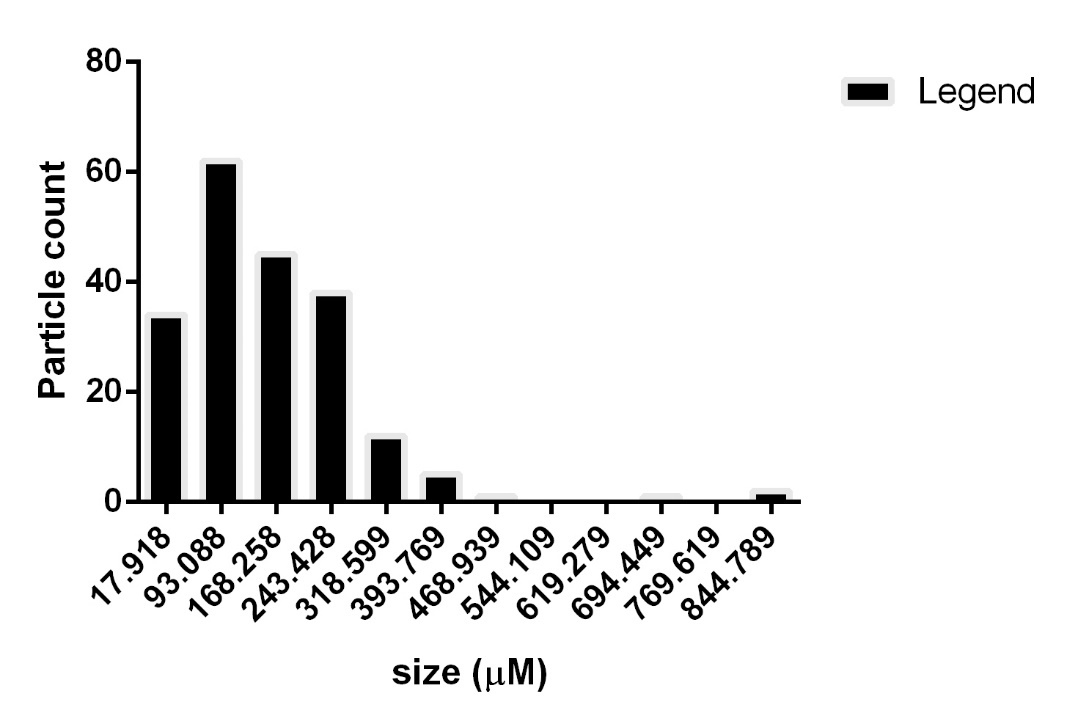


**Figure S8.** Histogram showing size distributions of the 125µm lower cut off fraction of black microplastic 2 (derived from Retsch solvent bottle lid). Mean size 194.39 µm ±123.12 SD. Sizes were measured manually on SEM images of >200 individual particles using ImageJ software.


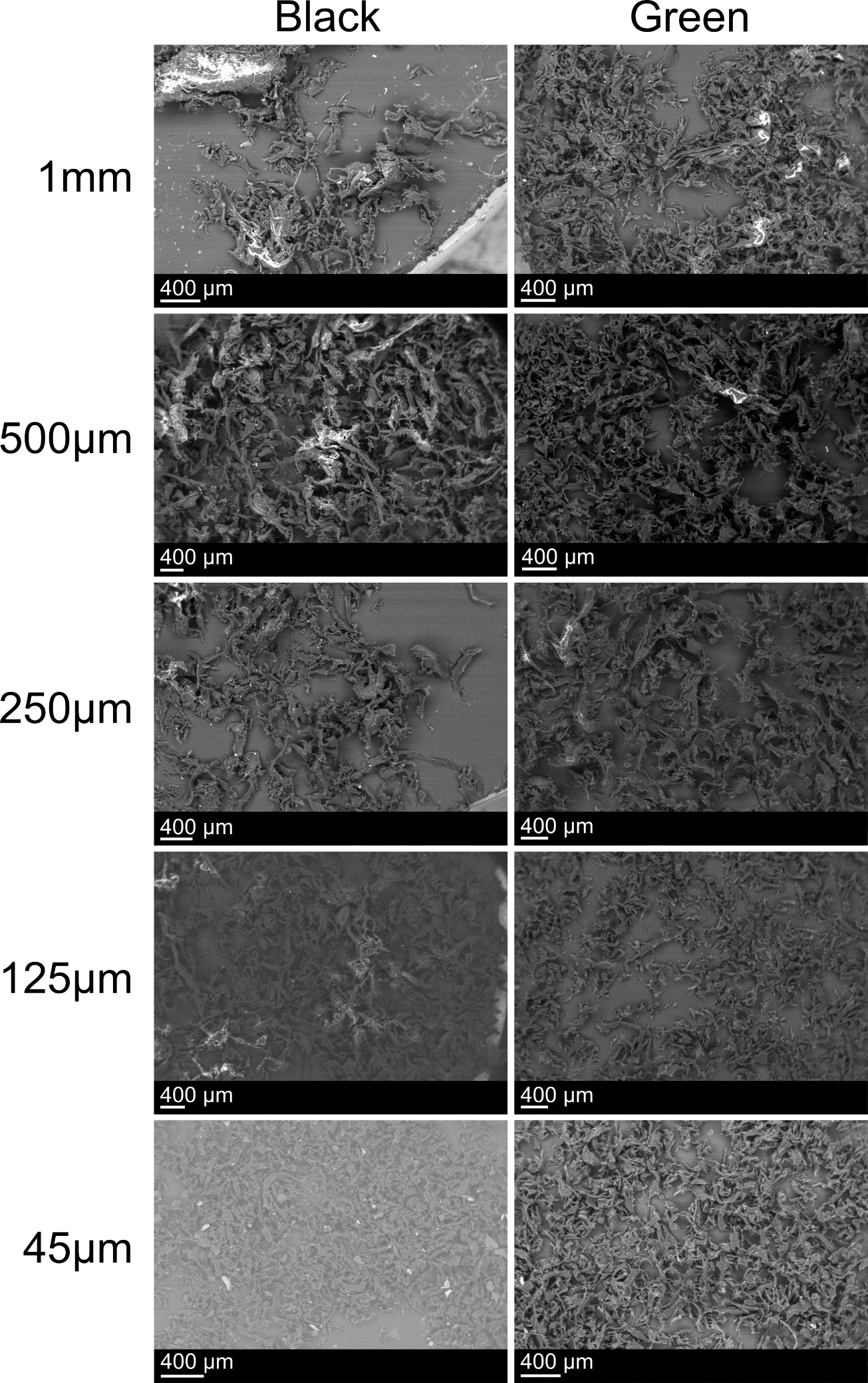


**Figure S9.** Scanning electron microscopy images of green (polyethylene, squash bottle lid) and black microplastic 2 (solvent bottle lid, polypropylene) samples post size-sieving. Size fractions are indicated on the left hand-side, and microplastic colour at the top.


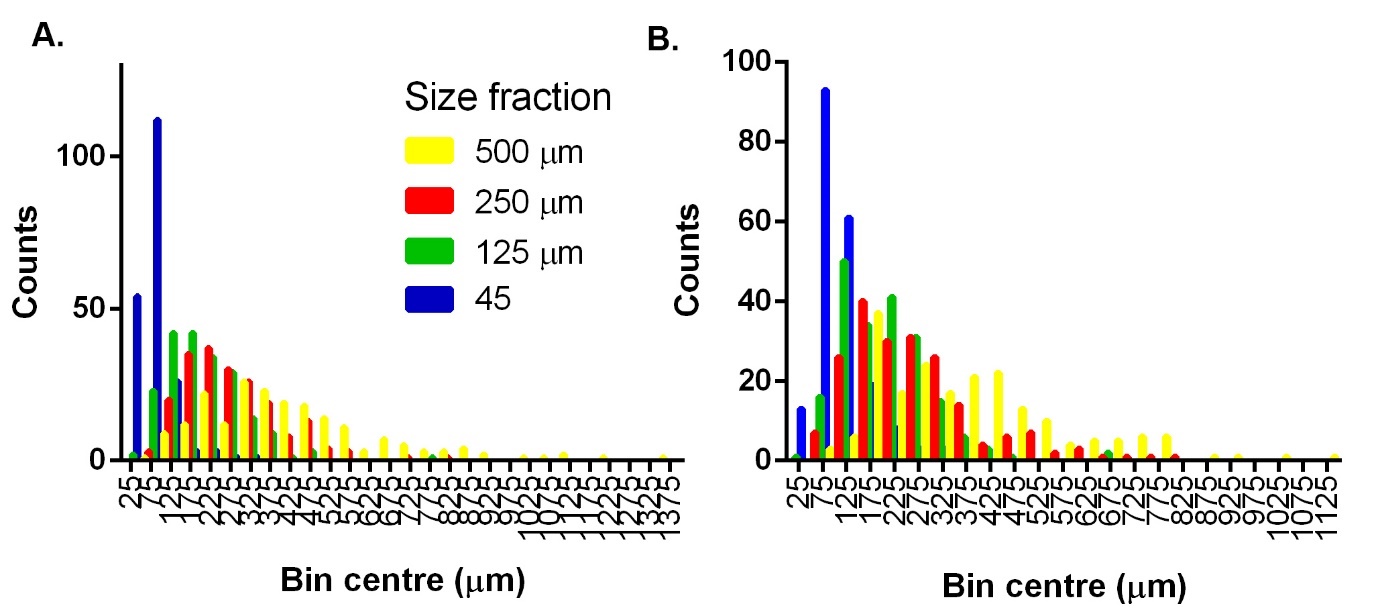


**Figure S10**. Histograms showing particle length distributions (n=200 particles measured in longest dimension per sieve fraction) from SEM of **A.** Black microplastic 2 (Polypropylene), and B. Green microplastic (Polyethylene). Mean, SD, and min and max particle sizes can be found in table S1.


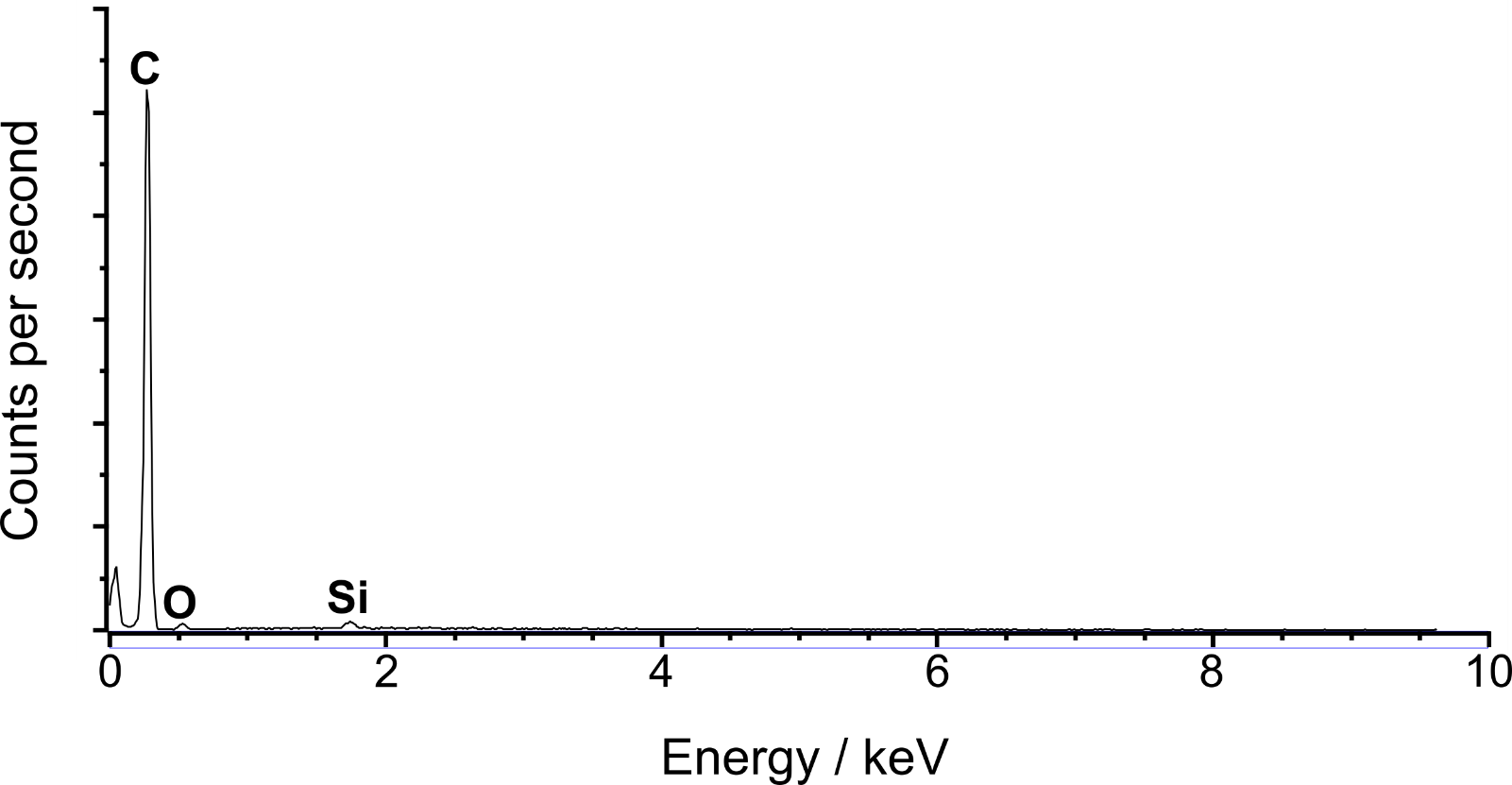


**Figure S11.** Representative energy dispersive X-ray spectrum (EDS) taken from the Black 500µm sample indicating the low proportion of silicon present. Composition results for all samples are summarised in Table S1.


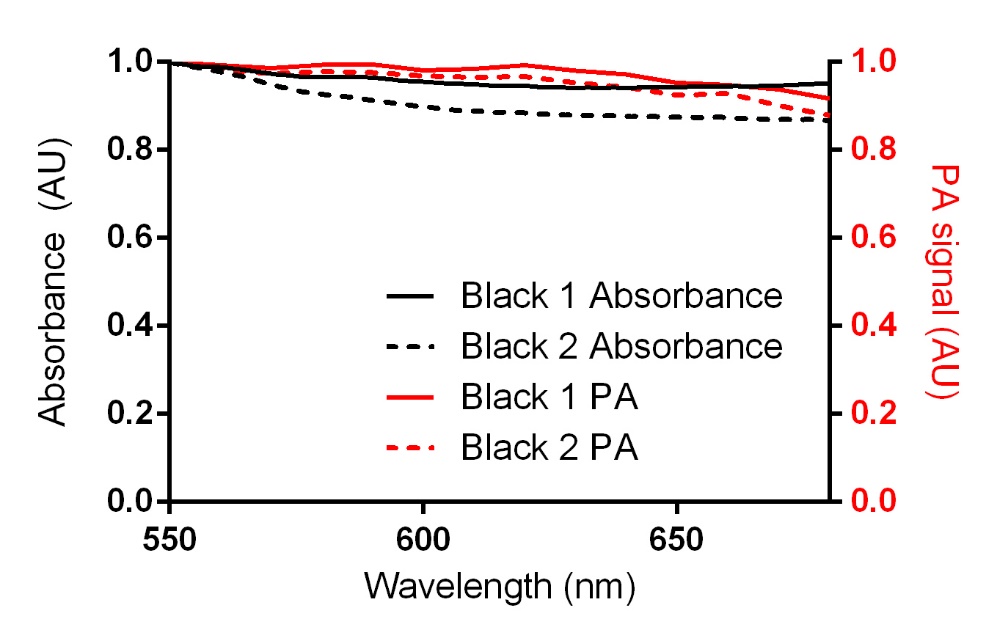


**Figure S12.** Photoacoustic spectrum acquired with 10nm resolution (see supplementary methods), using a 50mg/mL suspension of 125µm microplastics in water, overlaid with optical absorption spectrum.

**
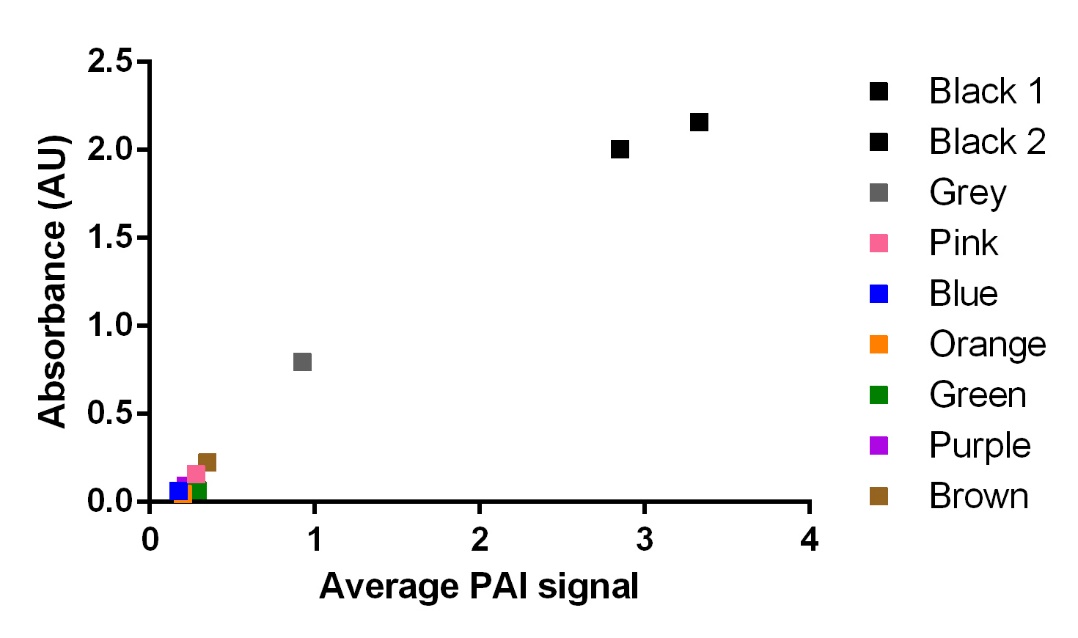
**

**Figure S13.** Absorbance at 980 nm of 9 microplastic samples (125µm fraction) plotted against their mean photoacoustic signal at 10 mg/mL in 2% agar with 980 nm illumination (using the VisualSonics LAZR-X system), showing a good correlation (R^2^=0.988).


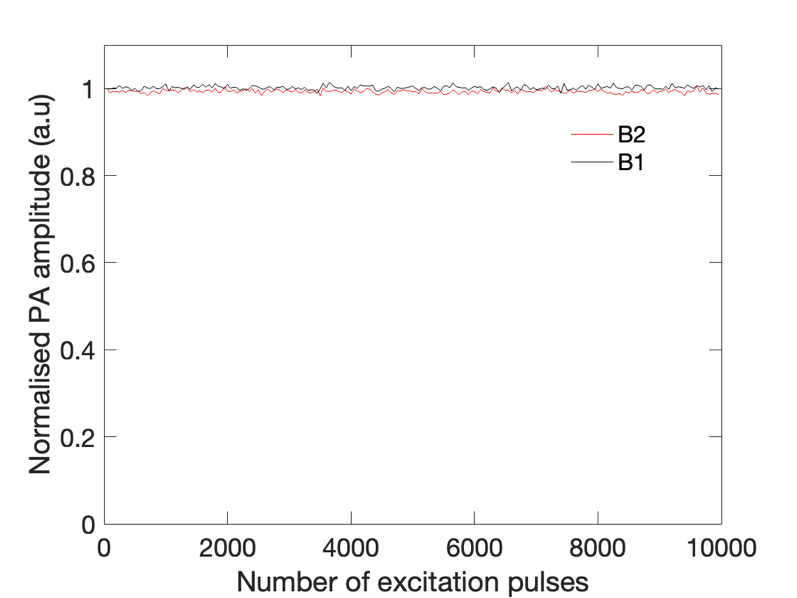


**Figure S14.** Normalized PA signal amplitude from two types of microplastic samples (Black 1, and Black 2) used in-vivo, under prolonged exposure to nanosecond laser pulses at 680nm. There is no reduction in the PA signal amplitude, indicating high photostability

**
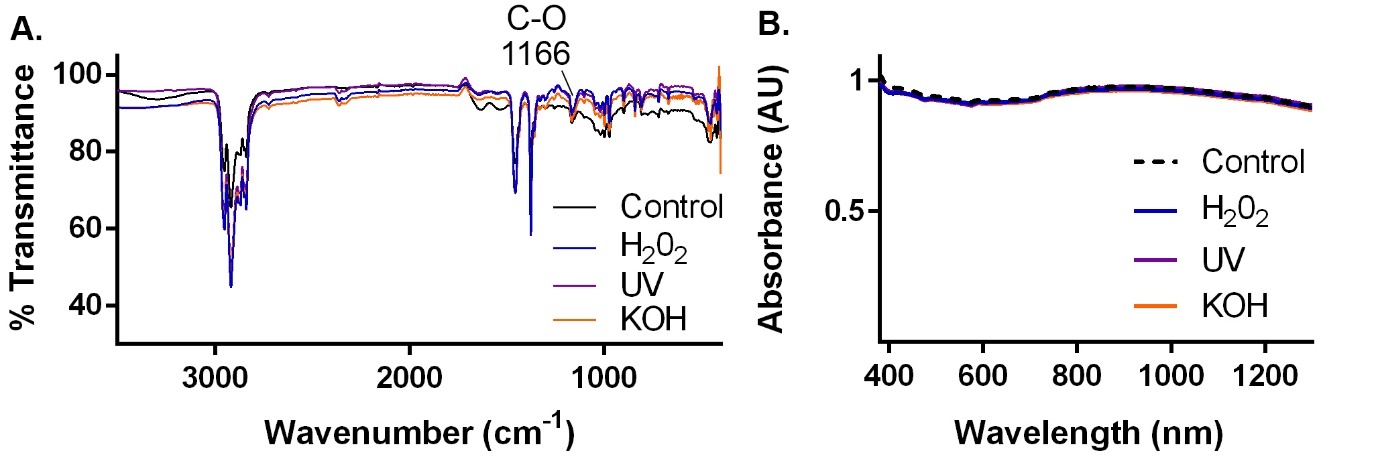
**

**Figure S15. A.** FTIR and **B.** Absorbance spectra of control (Black 250 µm polypropylene) and weathered samples show evidence of oxidation (C-O group formation), but no change in strength of optical absorbance. For weathering black 250 µm polypropylene microplastics were incubated for 1 week in 35% w/w H_2_O_2_ or 4M KOH before washing, or exposed to UV (254 nm, 6W, 96 hours), and characterised as above. Oxidation in the aged samples was confirmed via an increase in the C-O peak at 1166 cm^-1^ via ATR-FTIR.


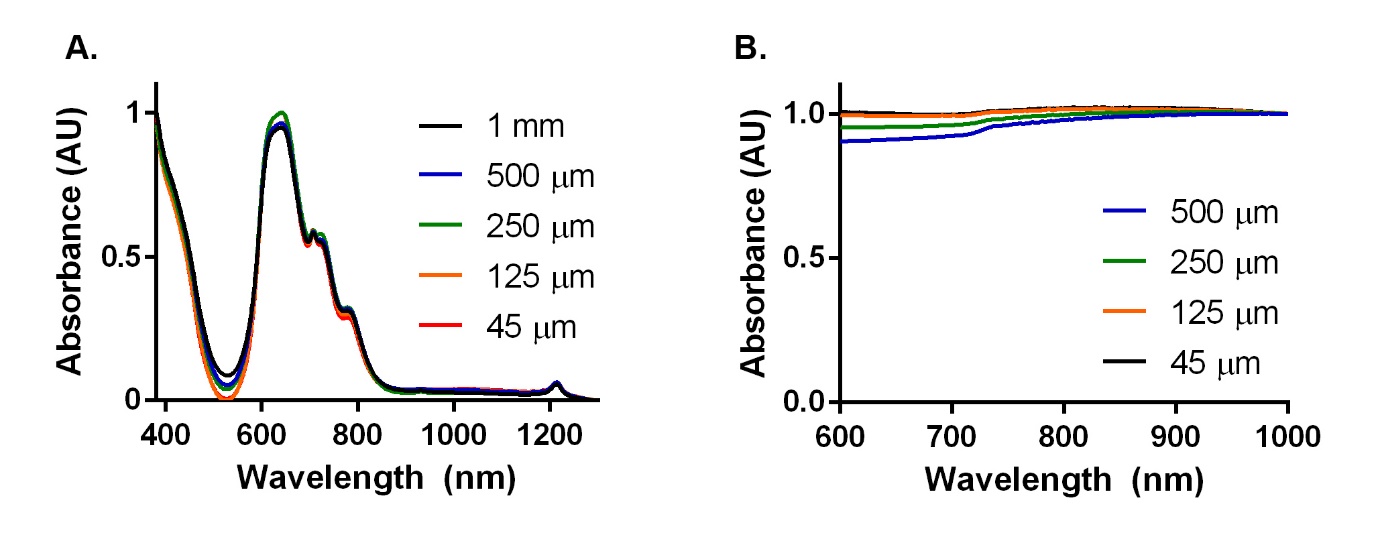
**Figure S16.** Normalised optical absorbance spectra for **A**. Green (polyethylene), and **B**. Black 2 (polypropylene) microplastics show no particle size-dependent changes.

**
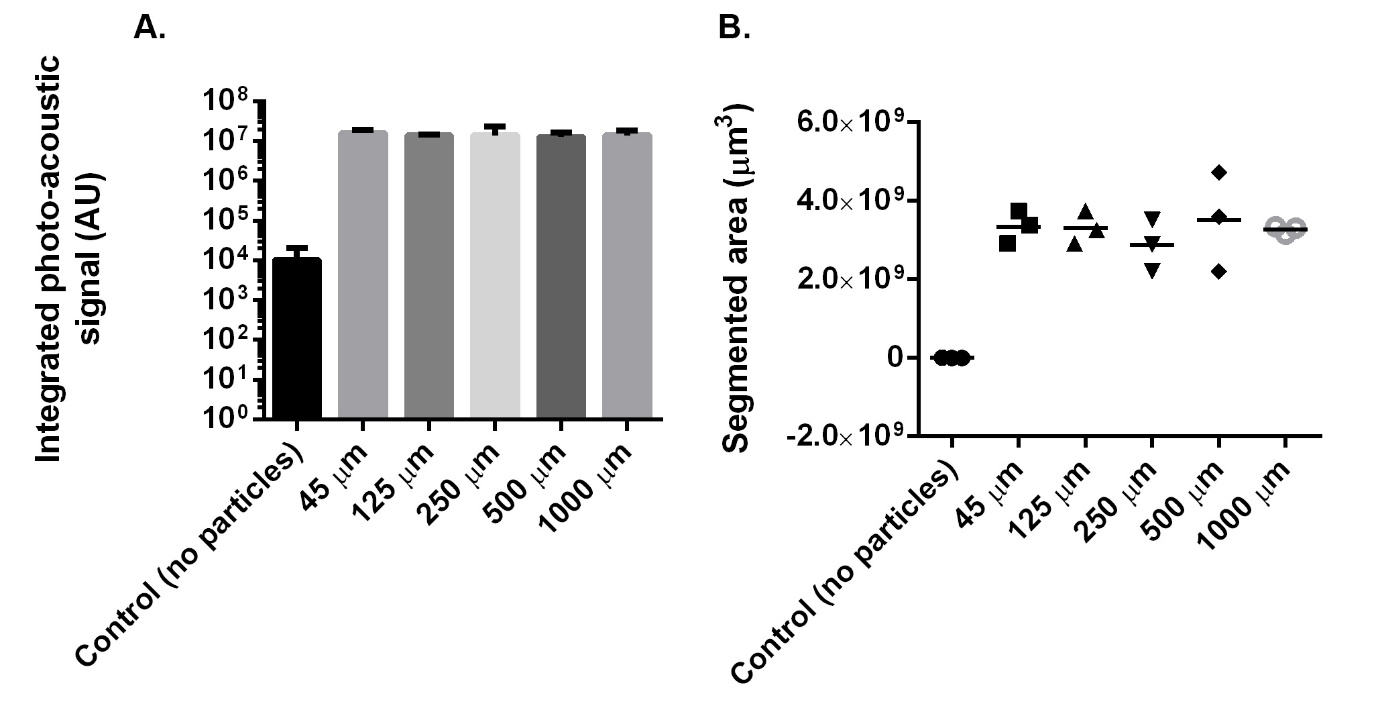
**

**Figure S17.** Quantification of **A.** integrated signal intensity, and **B**. segmented area showed no correlation with size (linear regression: R^2^ = 0.012, p=0.69, and R^2^=0.002, p=0.87 respectively) for grey polypropylene microplastics imaged at 980 nm (Deepcolor LightEcho-R) for a fixed weight of 1 mg.

**
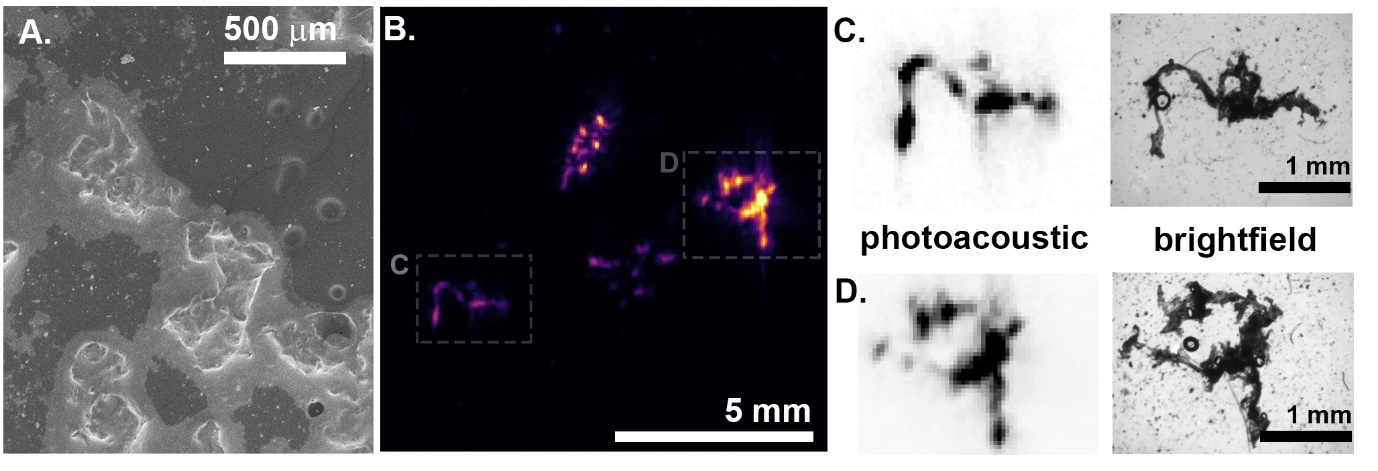
**

**Figure S18. Photoacoustic Imaging accurately reports microplastic structure on the microscale.** Representative **A.** Scanning electron micrograph, and **B.** Photoacoustic maximum intensity projection (MIP; 680nm illumination) of black microplastic 1 of black microplastic 1 (500 µm lower cut-off size fraction), with **C-D.** Enlarged views of the respective highlighted regions in B acquired with serial PAI and brightfield microscopy (inverted PAI grayscale to facilitate comparison). Pixelwise correlation analysis between modalities shown in C and D showed good agreement (Pearson’s correlation co-efficient C= 0.64, D=0.79).


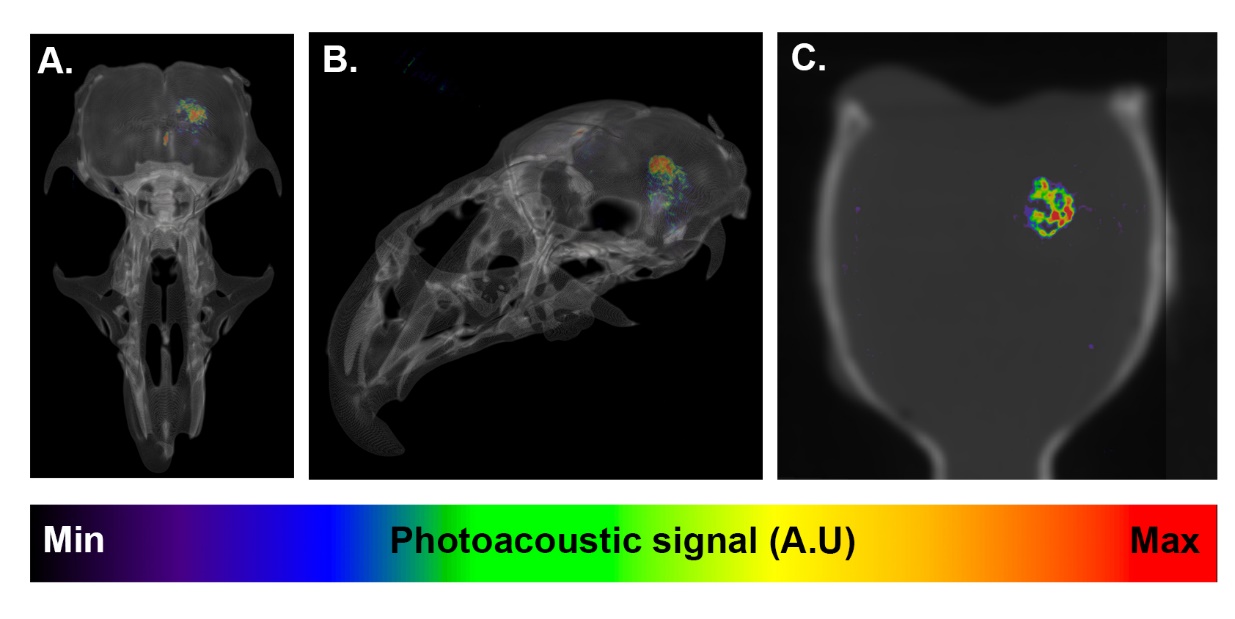


**Figure S19.** Representative Co-registered X-ray CT and Photoacoustic Image showing detection of black 1 microplastics through the rat skull (polypropylene, >125 µm <250 µm, 5mg/mL) as **A.** Maximum intensity Projection and **B.** Axial overlay. Microplastics were embedded in a 1% agar bead (right) with a control bead (left) in the contralateral hemisphere being undetectable above background.

**
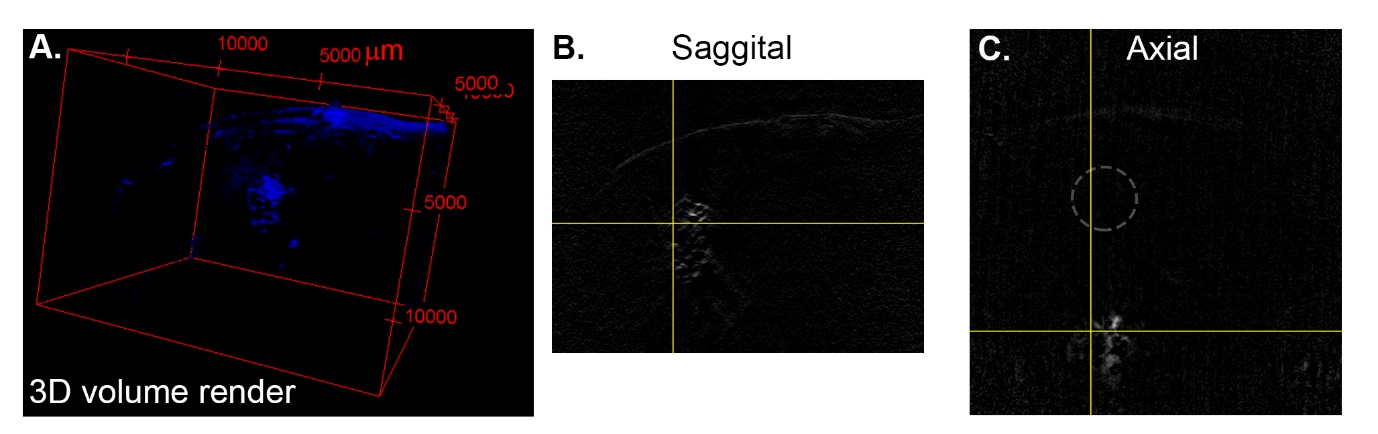
**

**Figure 20.** Representative photoacoustic images showing detection of blue microplastics through the rat skull (polypropylene, >125 µm <250 µm, 5mg/mL) as **A.** 3D volume render, **B.** Saggital and **C.** Axial single slice photoacoustic image (50 µm voxel depth, linear grayscale) showing hyper-intensity at the site of the microplastic containing bead. Dotted line shows site of control bead (agar only, no microplastic).

**
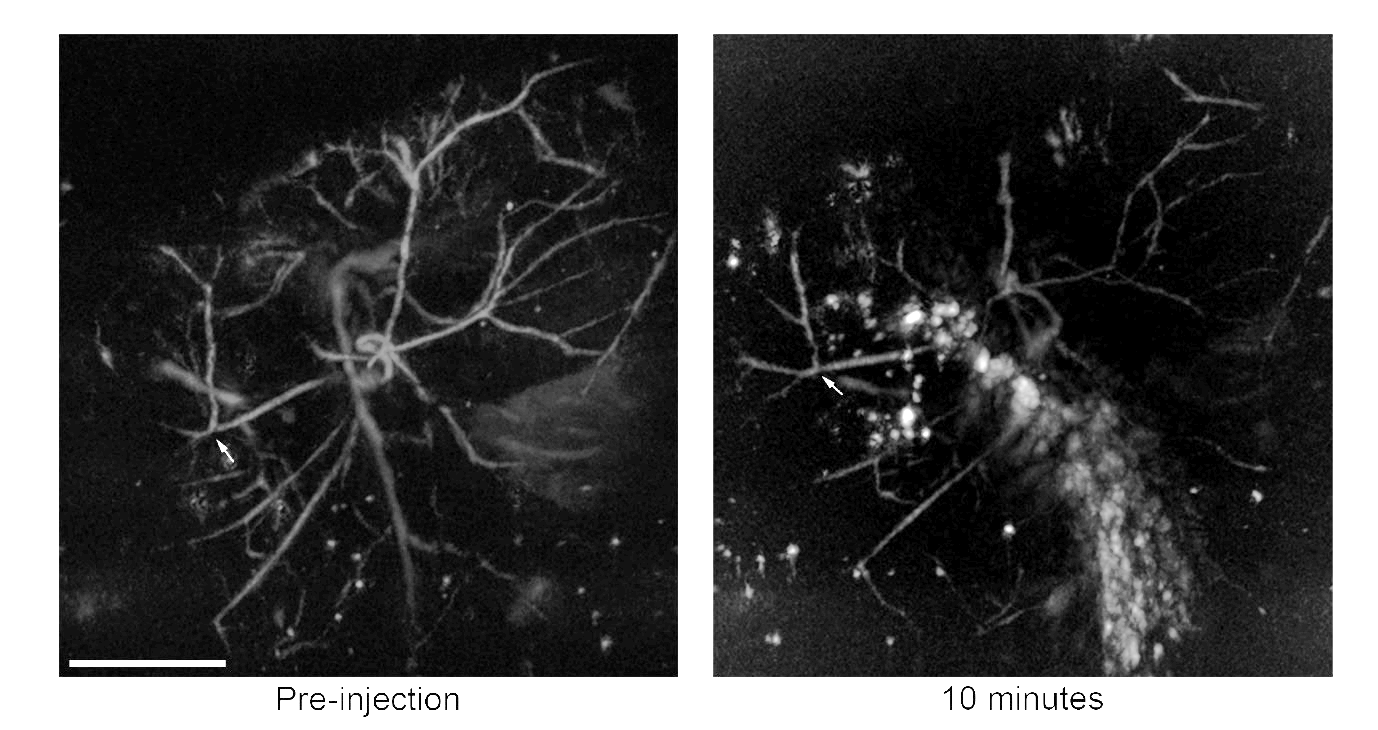
Figure S21.** Maximum intensity projections before (pre-injection), and ten minutes post subcutaneous injection of 0.5mg of blue polypropylene microplastics (>125 µm <250 µm). Photoacoustic images acquired over 4 averages using 680nm laser at 12mJ. Arrow indicates the same vascular feature on each image, provided for anatomical reference. Scale bar = 5mm

**
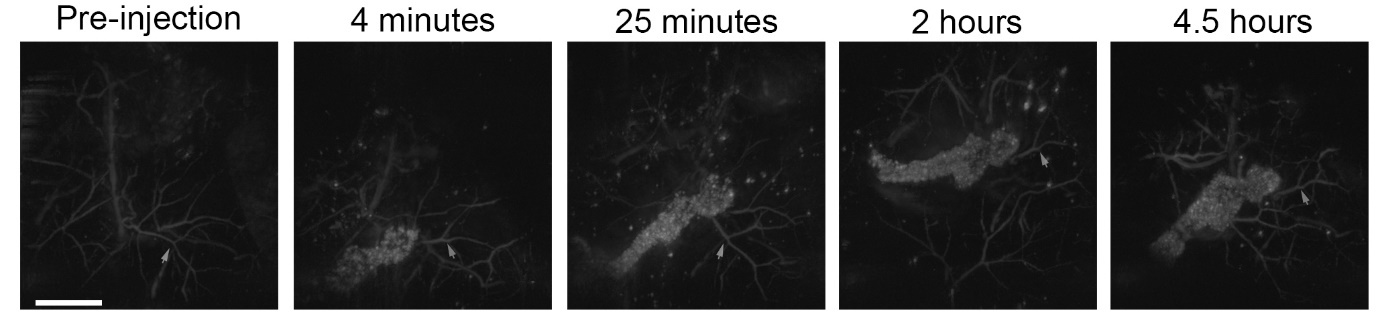
**

**Figure S22** Maximum intensity projections before (pre-injection), and up to four and a half hours post subcutaneous injection of 0.5mg of green polyethylene microplastics (>125 µm <250 µm). Photoacoustic images acquired over 4 averages using 680nm laser at 12mJ. Arrow indicates the same vascular feature on each image, provided for anatomical reference. Scale bar = 5mm


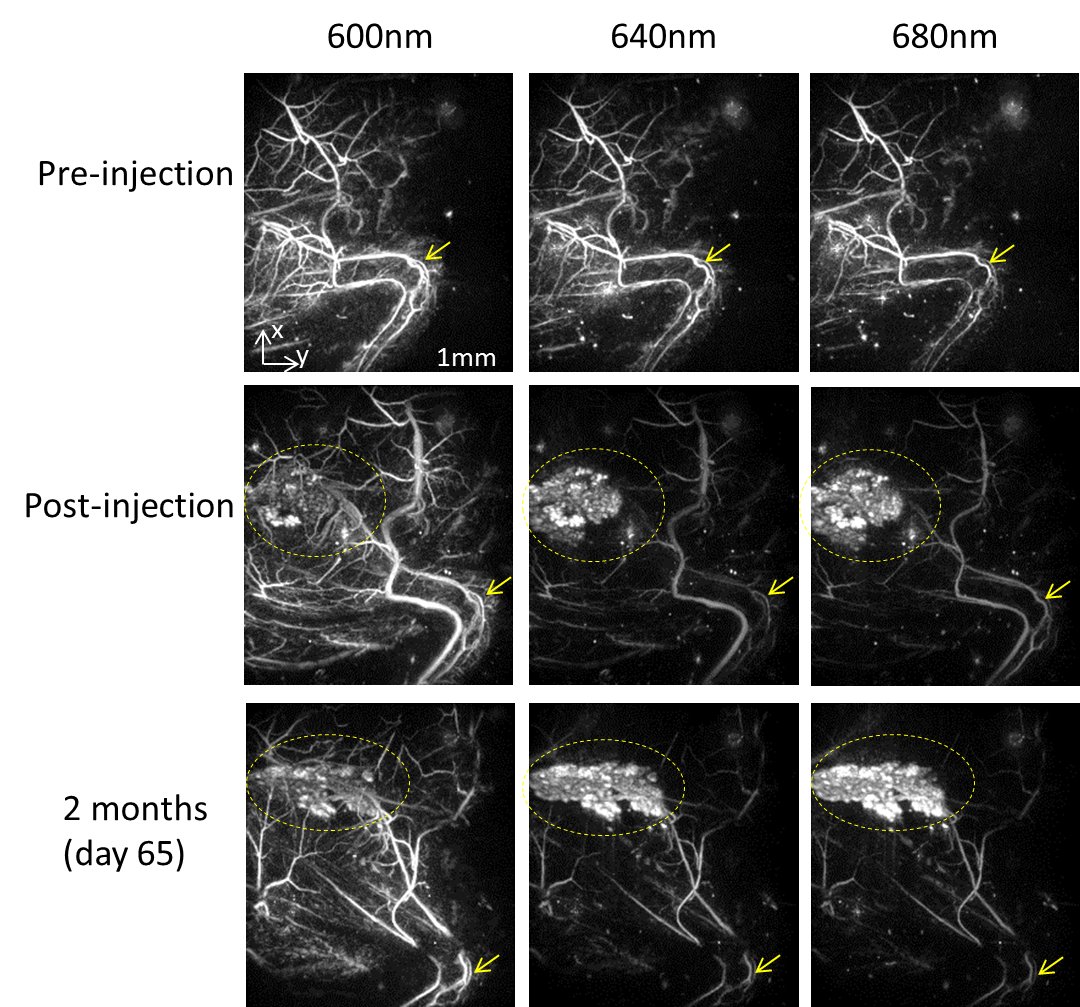


**Figure S23. Photoacoustic imaging of microplastics in the hind flank muscle.** Maximum intensity projections of the same region at 600, 640, and 680nm at pre-injection, 1 hour post injection (1mg microplastics), and 2 months (65 days) post injection. Yellow arrow points to the same reference vein to show image orientation


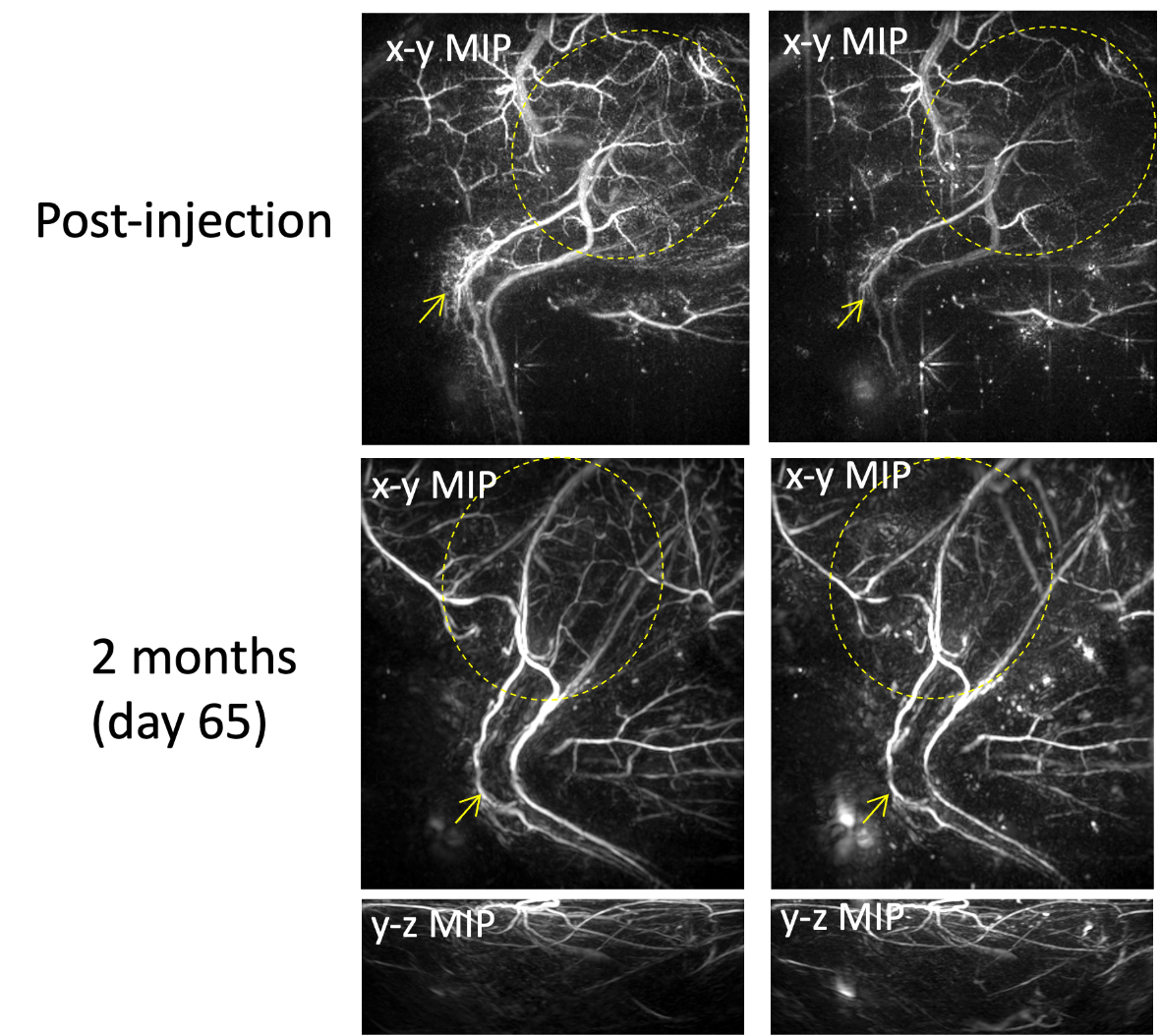


**Figure S24. Photoacoustic imaging shows absence of microplastics in the control (carrier only) hind flank muscle.** Maximum intensity projections of the same region at 600, and 680nm immediately after, and 2 months after injection of microplastics in the contralateral flank (shown in the previous supplementary figure). Control flank was injected with an equivalent volume of carrier only (60% Granugel in PBS). Yellow arrow points to the same reference vein to show image orientation.

|  | **500 µm** | **250 µm** | **125 µm** | **45 µm** |
| --- | --- | --- | --- | --- |
| **Green PE**  **(SD)**  **Min**  **Max** | 367.87  (191.22)  86.36  1112.38 | 269.41  (135.88)  68.75  805.70 | 205.38  (96.26)  38.84  692.08 | 109.77  (53.33)  22.10  324.62 |
| **Black PP 2**  **(SD)**  **Min**  **Max** | 431.28  (225.02)  91.41  1385.99 | 278.34  (124.35)  89.44  833.98 | 202.264  (98.57)  40.75  785.25 | 73.01  (40.06)  19.35  300.02 |
|  |  |  | 194.39  (123.12)  17.92  919.96 |  |
| **Black PP 1**  **(SD)**  **Min**  **Max** |  |  | 166.36  (68.23)  31.11  562.91 |  |

**Table S1.** Particle length measurements (longest dimension) derived from SEM analysis for green and black 2 microplastic batches across the fractions retained in the indicated sieve sizes (n=200 per batch).

|  | **Composition / atomic %** | | | | |
| --- | --- | --- | --- | --- | --- |
| **Sample name** | **Carbon** | **Oxygen** | **Silicon** | **Sulfur** | **Calcium** |
| Green 1mm | 95.33 | 4.48 | 0.11 | 0.08 | ­ |
| Green 500um | 95.04 | 4.70 | 0.10 | 0.11 | 0.06 |
| Green 250um | 95.35 | 4.51 | 0.06 | 0.08 | ­ |
| Green 125um | 93.42 | 6.38 | 0.09 | 0.11 | ­ |
| Green 45um | 96.91 | 2.94 | 0.10 | ­ | 0.05 |
|  |  |  |  |  |  |
| Black 1mm | 94.24 | 5.62 | 0.14 | ­ | ­ |
| Black 500 um | 96.18 | 3.57 | 0.24 | ­ | ­ |
| Black 250um | 94.47 | 5.26 | 0.19 | 0.08 | ­ |
| Black 125um | 94.82 | 4.93 | 0.20 | 0.05 | ­ |
| Black 45um | 92.66 | 6.98 | 0.30 | 0.06 | ­ |
|  |  |  |  |  |  |
| **Average comp:** | 94.842 | 4.937 | 0.153 | 0.081429 | 0.055 |

**Table S2**: Atomic % composition taken from energy dispersive X-ray spectra (EDS) from different microplastic size fractions. Note the persistent presence of silicon in all samples at an average composition of 0.153%.

**Supplementary References**

1. Stahl T, Allen T, Beard P. Characterization of the thermalisation efficiency and photostability of photoacoustic contrast agents. Photons Plus Ultrasound: Imaging and Sensing 20142014.

2. Schindelin J, Arganda-Carreras I, Frise E, Kaynig V, Longair M, Pietzsch T, et al. Fiji: an open-source platform for biological-image analysis. Nat Methods. 2012;9(7):676-82.
